# Optineurin promotes myogenesis during muscle regeneration in mice by autophagic degradation of GSK3β

**DOI:** 10.1371/journal.pbio.3001619

**Published:** 2022-04-27

**Authors:** Xiao Chen Shi, Bo Xia, Jian Feng Zhang, Rui Xin Zhang, Dan Yang Zhang, Huan Liu, Bao Cai Xie, Yong Liang Wang, Jiang Wei Wu

**Affiliations:** Key Laboratory of Animal Genetics, Breeding and Reproduction of Shaanxi Province, College of Animal Science and Technology, Northwest A&F University, Yangling, China; King’s College London, UNITED KINGDOM

## Abstract

Skeletal muscle regeneration is essential for maintaining muscle function in injury and muscular disease. Myogenesis plays key roles in forming new myofibers during the process. Here, through bioinformatic screen for the potential regulators of myogenesis from 5 independent microarray datasets, we identify an overlapping differentially expressed gene (DEG) optineurin (OPTN). *Optn* knockdown (KD) delays muscle regeneration in mice and impairs C2C12 myoblast differentiation without affecting their proliferation. Conversely, *Optn* overexpression (OE) promotes myoblast differentiation. Mechanistically, OPTN increases nuclear levels of β-catenin and enhances the T-cell factor/lymphoid enhancer factor (TCF/LEF) transcription activity, suggesting activation of Wnt signaling pathway. The activation is accompanied by decreased protein levels of glycogen synthase kinase 3β (GSK3β), a negative regulator of the pathway. We further show that OPTN physically interacts with and targets GSK3β for autophagic degradation. Pharmacological inhibition of GSK3β rescues the impaired myogenesis induced by *Optn* KD during muscle regeneration and myoblast differentiation, corroborating that GSK3β is the downstream effector of OPTN-mediated myogenesis. Together, our study delineates the novel role of OPTN as a potential regulator of myogenesis and may open innovative therapeutic perspectives for muscle regeneration.

## Abstract 2

Skeletal muscle regeneration is essential for maintaining muscle function in injury and muscular disease. This study identifies optineurin as a promoter of myoblast differentiation and potential regulator of myogenesis, showing that it interacts with GSK3β and targets it for autophagic degradation.

## Introduction

Skeletal muscle, the most abundant tissue in our bodies, plays key roles in posture, mobility, and energy metabolism [1]. In muscle injury and muscular disease, the regeneration capacity of skeletal muscle is essential for restoration of these functions [2,3]. In response to muscle injury, satellite cells (SCs) will be activated to start myogenic differentiation, accompanied with up-regulated expression of myogenin (MYOG) and muscle-specific regulatory factor 4 [4,5]. The differentiation program is then completed with the activation of muscle specific proteins such as MYHC in myoblasts that subsequently fuse to regenerate myofibers to repair damaged muscle [2]. Therefore, myoblast differentiation-mediated myogenesis plays essential roles in muscle regeneration. Nonetheless, the underlying mechanisms of myogenesis during muscle regeneration remain largely unknown.

To explore the potential regulators of myogenesis, we performed bioinformatics screen from 5 myogenesis-related microarray datasets and identified optineurin (*Optn*) as one of the 5 overlapping genes, up-regulated during myoblast differentiation and muscle regeneration, while down-regulated in Duchenne muscular dystrophy (DMD) patients and mdx mice. The human OPTN is a 74-kDa scaffold protein comprised of 577 amino acids, and the mouse *Optn* gene encodes for a 584-amino acid protein (67 kDa), which is 78% identical to human OPTN [6]. OPTN is expressed in most tissues, including muscle, liver, and brain [7–9], and plays important roles in many cellular functions [6]. It has been identified as a selective autophagy receptor involved in the various stages of the autophagic process such as cargo recognition, autophagosome formation, and autophagic degradation [10]. *OPTN* mutations were shown in several familial diseases and often occur in its autophagy-associated ubiquitin-binding domain (UBAN) [11], such as *OPTN* ^E478G^ in amyotrophic lateral sclerosis (ALS) [7] and *OPTN* ^R545Q^ in normal-tension glaucoma [12]. OPTN is highly expressed in the skeletal muscle [13], yet little is known about the role of OPTN in myogenesis and whether its function in skeletal muscle is related to autophagy.

The canonical Wnt signaling pathway plays critical roles in facilitating the differentiation of SCs during skeletal muscle regeneration [14–16]. Wnt ligands bind to frizzled receptors and members of the low-density lipoprotein receptor related protein family, activating the nuclear translocation of β-catenin and the formation of a complex with the T-cell factor/lymphoid enhancer factor (TCF/LEF) [17]. It enhances transcriptional activity of myogenic factors such as myogenic factor 5 (*Myf5*) [18], myoblast determination protein (*Myod*) [19], fermitin family homolog 2 (*Fermt2*) [20], and *Myog* [21] during muscle regeneration. Wnts promote β-catenin nuclear translocation through inhibition of glycogen synthase kinase 3β (GSK3β), an important component of β-catenin destruction complex [22]. Genetic deletion or pharmacological inhibition of GSK3β leads to enhanced differentiation of C2C12 cells and muscle regeneration [23,24], indicating that GSK3β is essential for Wnt signaling pathway–mediated myoblast differentiation. Nevertheless, mechanism of Wnt-mediated suppression of GSK3β remains incompletely resolved and in dispute. A GSK3β-mediated crosstalk of autophagy and canonical Wnt signaling pathway has been shown during embryogenesis [25–27]. Whether the autophagy receptor OPTN is involved in the GSK3β-mediated canonical Wnt signaling pathway during muscle regeneration is completely unknown.

In this study, we show that OPTN is required for myoblast differentiation-mediated myogenesis during muscle regeneration in mice. OPTN promotes Wnt signaling pathway mediated myogenesis through direct physical interaction and autophagic degradation of GSK3β. Our findings reveal a new insight into mechanism underlying myogenesis during muscle regeneration and provide a potential target for muscle regeneration.

## Results

### Bioinformatic screen reveals OPTN as a potential regulator for myogenesis

**Fig 1:**
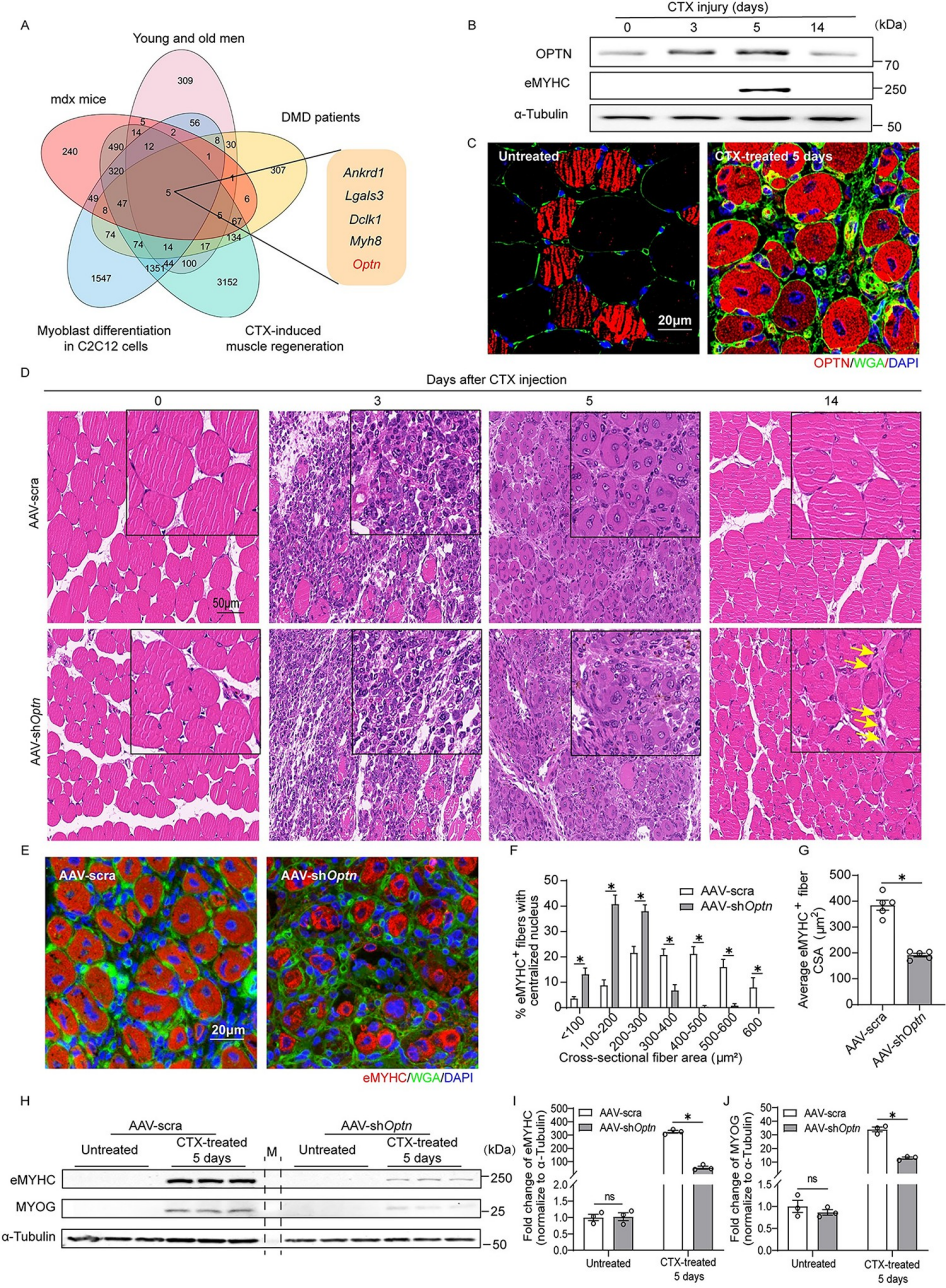
OPTN is essential for myogenesis during skeletal muscle regeneration in response to muscle injury. **(A)** Venn diagram showing 5 overlapping DEGs among 5 independent microarray datasets related to myogenesis or muscle atrophy. **(B)** Representative immunoblotting analysis of eMYHC and OPTN in TA of WT mice at 0, 3, 5, and 14 days postinjury (*n* = 3 mice in each group). **(C)** Representative immunofluorescence analysis of OPTN subcellular distribution in TA muscle at CTX untreated or 5 days postinjury. The OPTN, the sarcolemma, and the nucleus were stained with anti-OPTN antibody (red), WGA (green), and DAPI (blue), respectively. Scale bars: 20 um. **(D)** Representative HE staining of TA at 0, 3, 5, and 14 days postinjury in scramble shRNA or sh*Optn* mice. The yellow arrowheads indicate inflammatory cells infiltration. Scale bar: 50 μm. **(E)** Representative immunofluorescence staining of eMYHC^+^ fibers in scramble shRNA or sh*Optn* TA muscle at 5 days postinjury. Scale bar: 20 μm. **(F)** Distribution of eMYHC^+^ myofiber CSAs in scramble shRNA or sh*Optn* TA muscle at 5 days postinjury (*n* = 5 mice in each group). (G) Average CSA of regenerating eMYHC^+^ myofibers in scramble shRNA or sh*Optn* TA muscle at 5 days postinjury (*n* = 5 mice in each group). (H–J) Representative immunoblotting analysis (H) and quantification (I, J) of myogenic markers (eMYHC and MYOG) in scramble shRNA or sh*Optn* TA muscle (*n* = 3 mice in each group) at 5 days postinjury. M, marker. Data are presented as mean ± SEM. *P < 0.05 versus control. The underlying data for this figure can be found in **S1 Data**. The original blot for this figure can be found in **S1 Raw Image**. AAV, adeno-associated viral vector; Ankrd1, ankyrin repeat domain 1; CTX, cardiotoxin; CSA, cross-sectional fiber area; Dclk1, doublecortin like kinase 1; DEG, differentially expressed gene; DMD, Duchenne muscular dystrophy; eMYHC, embryonic myosin heavy chain; HE, hematoxylin–eosin; Lgals3, galectin 3; mdx, murine X-linked muscular dystrophy; Myh8, myosin heavy chain 8; MYOG, myogenin; Optn, optineurin; SEM, standard error of the mean; shRNA, short hairpin RNA; TA, tibialis anterior; WGA, wheat germ agglutinin; WT, wild-type.

To search for potential regulators of myogenesis, we compiled and intercrossed differentially expressed genes (DEGs) in 5 independent microarray datasets related to myogenesis from the Gene Expression Omnibus (GEO) database: (i) DEGs during C2C12 cell differentiation (GSE11415); (ii) DEGs during cardiotoxin (CTX)-induced muscle regeneration in mice (GSE45577); (iii) DEGs in skeletal muscle of DMD patients (GSE1004); (iv) DEGs in gastrocnemius muscle of murine X-linked muscular dystrophy (mdx) mice (GSE16438); and (v) DEGs in vastus lateralis between young (21 to 31 years old) and old (62 to 77 years old) men (GSE80). Comprehensive analysis of the datasets yielded 5 overlapping genes ankyrin repeat domain 1 (*Ankrd1*), galectin 3 (*Lgals3*), doublecortin like kinase 1 (*Dclk1*), myosin heavy chain 8 (*Myh8*), and *Optn* (Fig 1A). Among them, *Ankrd1*, *Lgals3*, *Dclk1*, and *Myh8* have been well characterized in the regulation of myogenesis [28–32]. The roles of OPTN, a selective autophagy receptor [33], remain unclear in myogenesis.

### OPTN is essential for myogenesis during skeletal muscle regeneration

To investigate the role of OPTN in myogenesis during muscle regeneration, we analyzed OPTN expression during CTX-induced muscle injury in mice. Compared with uninjured muscle (day 0), OPTN expression was significantly up-regulated during the initial phase of muscle regeneration (day 5), and then decreased at day 14, similar to the expression profile of the newly regenerated myofiber marker embryonic myosin heavy chain (eMYHC) (Fig 1B, S1A and S1B Fig). In line with this, the immunofluorescence analysis also showed increased OPTN in the cytoplasm of newly regenerated myofibers at 5 days postinjury (Fig 1C, S2 Fig). These results indicate a potential role of OPTN in muscle regeneration. We further generated a recombinant adeno-associated viral vector (AAV) with a short hairpin RNA (shRNA) targeting *Optn* (AAV-sh*Optn*) (S3A and S3B Fig), which achieved 72% reduction in mRNA levels and 76% reduction in protein levels compared with scramble shRNA in tibialis anterior (TA) muscle (S3C and S3D Fig). The TA muscle receiving AAV scramble shRNA or sh*Optn* was subjected to a single CTX injury and then allowed to recover for 3 to 14 days before analysis of the regenerated tissue. In scramble shRNA muscle, SCs descendants fused to form new myofibers characterized by centrally localized nucleus during the acute phase of regeneration (3 to 5 days after injury) (Fig 1D). The eMYHC^+^ fibers were abundant at day 5 postinjury (Fig 1E). In contrast, AAV-sh*Optn* muscle was composed of degenerating myofibers, fibrotic tissues, and inflammatory cells at this phase (Fig 1D). The eMYHC^+^ regenerating fibers in AAV-sh*Optn* muscle were less and smaller at day 5 postinjury compared with scramble shRNA muscle (Fig 1E–1G). In line with this, the protein levels of eMYHC and MYOG were significantly reduced in AAV-sh*Optn* muscle at day 5 postinjury (Fig 1H–1J). Fourteen days after injury, muscle damage and inflammatory cells in scramble shRNA muscle were largely cleared, and the regenerated myofibers continued to grow and mature, as they became homogenous in size (Fig 1D), whereas small regenerated fibers and a few inflammatory cells were still shown in sh*Optn* TA muscle (Fig 1D). These data show that *Optn* knockdown (KD) delayed skeletal muscle regeneration in adult mice, indicating an essential role of OPTN in myogenesis during muscle regeneration.

### OPTN promotes myoblast differentiation-mediated myogenesis

**Fig 2:**
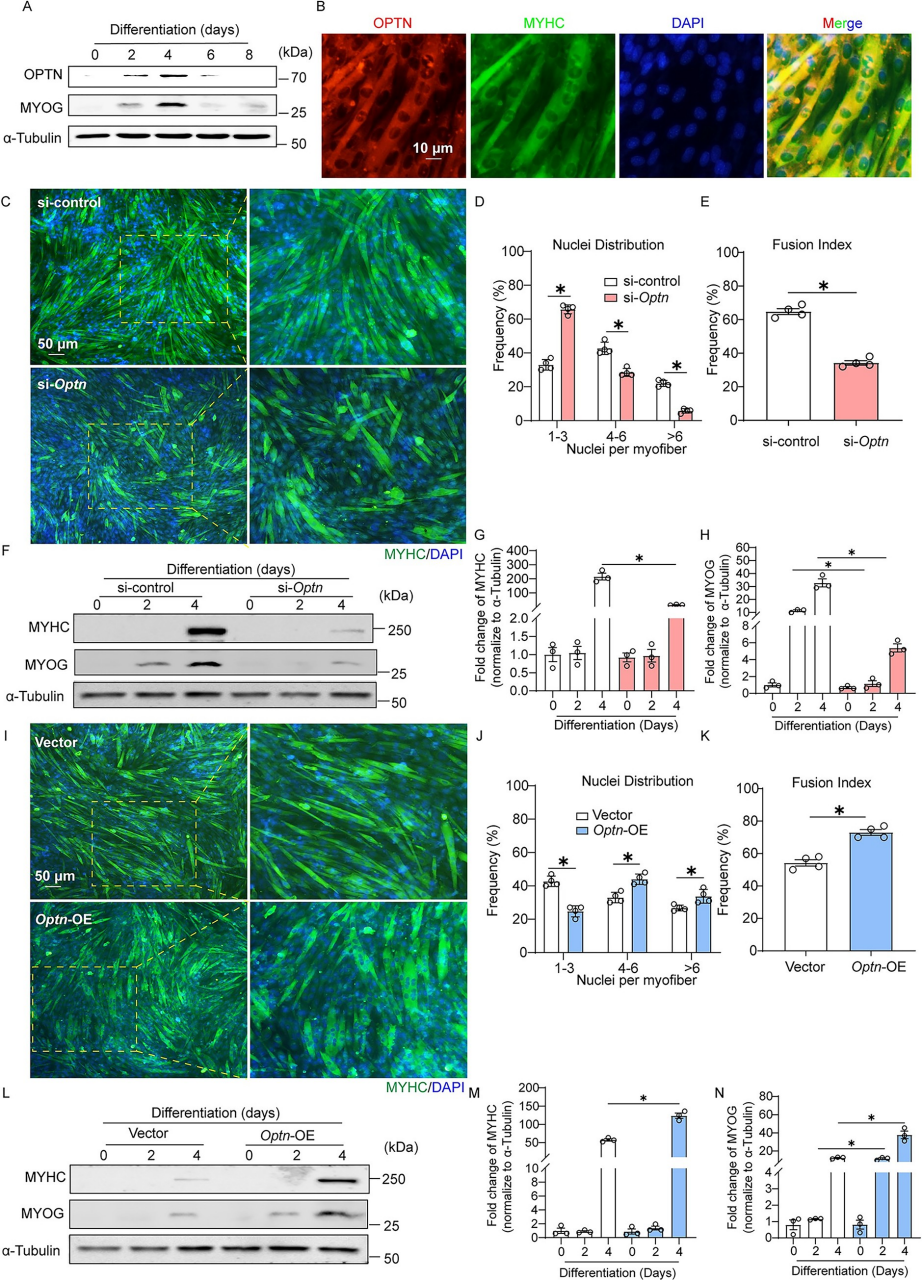
OPTN promotes myoblast differentiation mediated myogenesis. **(A)** Representative immunoblotting analysis of OPTN and MYOG in C2C12 cells during differentiation at the indicated time points (0, 2, 4, 6, and 8 days) (*n* = 3 in each group). **(B)** Representative immunofluorescence analysis of OPTN subcellular distribution in myotubes. The OPTN and MYHC were stained with anti-OPTN antibody and anti-MYHC antibody in C2C12 cells at 4 days postdifferentiation. Scale bars: 10 um. **(C)** Representative immunofluorescence staining of MYHC in control (si-control) or *Optn* KD (si-*Optn*) C2C12 cells at 4 days postdifferentiation. si-control or si-*Optn* were transfected into C2C12 cells for 48 hours before the initiation of differentiation. Scale bars: 50 μm. **(D, E)** Quantification of the nucleus distribution per myotube and fusion index (a MYHC^+^ cell with at least 3 nucleus) in control (si-control) or *Optn* KD (si-*Optn*) C2C12 cells at 4 days postdifferentiation (*n* = 4 in each group). **(F–H)** Representative immunoblotting analysis (F) and quantification (G, H) of MYHC and MYOG in control (si-control) or *Optn* KD (si-*Optn*) C2C12 cells (*n* = 3 in each group). Cells were collected at 0, 2, and 4 days postdifferentiation, respectively. **(I)** Representative immunofluorescence staining of MYHC in control (empty vector) or *Optn*-OE C2C12 cells at 4 days postdifferentiation. The empty pcDNA 3.1-HA vector or pcDNA 3.1-HA-*Optn* vector were transfected into C2C12 cells for 48 hours before the initiation of differentiation. Scale bars: 50 μm. **(J, K)** Quantification of the distribution of nucleus per myotube and the fusion index (a MYHC^+^ cell with at least 3 nucleus) in control (empty vector) or *Optn*-OE C2C12 cells at 4 days postdifferentiation (*n* = 4 in each group). **(L–N)** Representative immunoblotting analysis (L) and quantification (M, N) of MYHC and MYOG in control (empty vector) or *Optn*-OE C2C12 cells (*n* = 3 in each group). Cells were collected at 0, 2, and 4 days postdifferentiation, respectively. Data are presented as mean ± SEM. *P < 0.05 versus control. The underlying data for this figure can be found in **S1 Data**. The original blot for this figure can be found in **S1 Raw Image**. KD, knockdown; MYOG, myogenin; MYHC, myosin heavy chain; OE, overexpressing; OPTN, optineurin; SEM, standard error of the mean.

In response to muscle injury, muscle SCs undergo massive proliferation and differentiation to form new myotubes that replace the damaged myofibers [34]. To explore the role of OPTN in myogenesis during muscle regeneration, we first detected the effect of OPTN on muscle SCs proliferation and found similar paired box 7^+^ (Pax7) 5-ethynyl-20-deoxyuridine^+^ (EdU) SCs frequency in sh*Optn* and scramble shRNA TA muscle at day 3 postinjury (S4A–S4D Fig). In addition, *Optn* KD in C2C12 cells had no effect on numbers of EdU^+^ cells and the expression of cell proliferation-associated genes (S4E–S4G Fig). These results suggest that OPTN does not affect SCs proliferation during muscle regeneration. We next investigated whether OPTN-mediated myogenesis is achieved by regulation of myoblast differentiation. OPTN expression was increased during C2C12 myoblast differentiation with MYHC colocalization, similar to the expression patterns of MYOG (Fig 2A and 2B, S5A and S5B Fig). *Optn* KD in C2C12 cells reduced cell fusion and multinuclear myotube formation events (Fig 2C–2E), along with decreased levels of MYOG and MYHC (Fig 2F–2H, S6A and S6B Fig). In contrast, *Optn* overexpression (OE) (plasmid HA-*Optn*) in C2C12 cells dramatically enhanced myoblast differentiation (Fig 2I–2K), accompanied with increased levels of MYOG and MYHC (Fig 2L–2N, S6C and S6D Fig). Together, these findings indicate that OPTN promotes myoblast differentiation-mediated myogenesis.

### OPTN enhances myogenesis through activation of canonical Wnt signaling pathway

**Fig 3:**
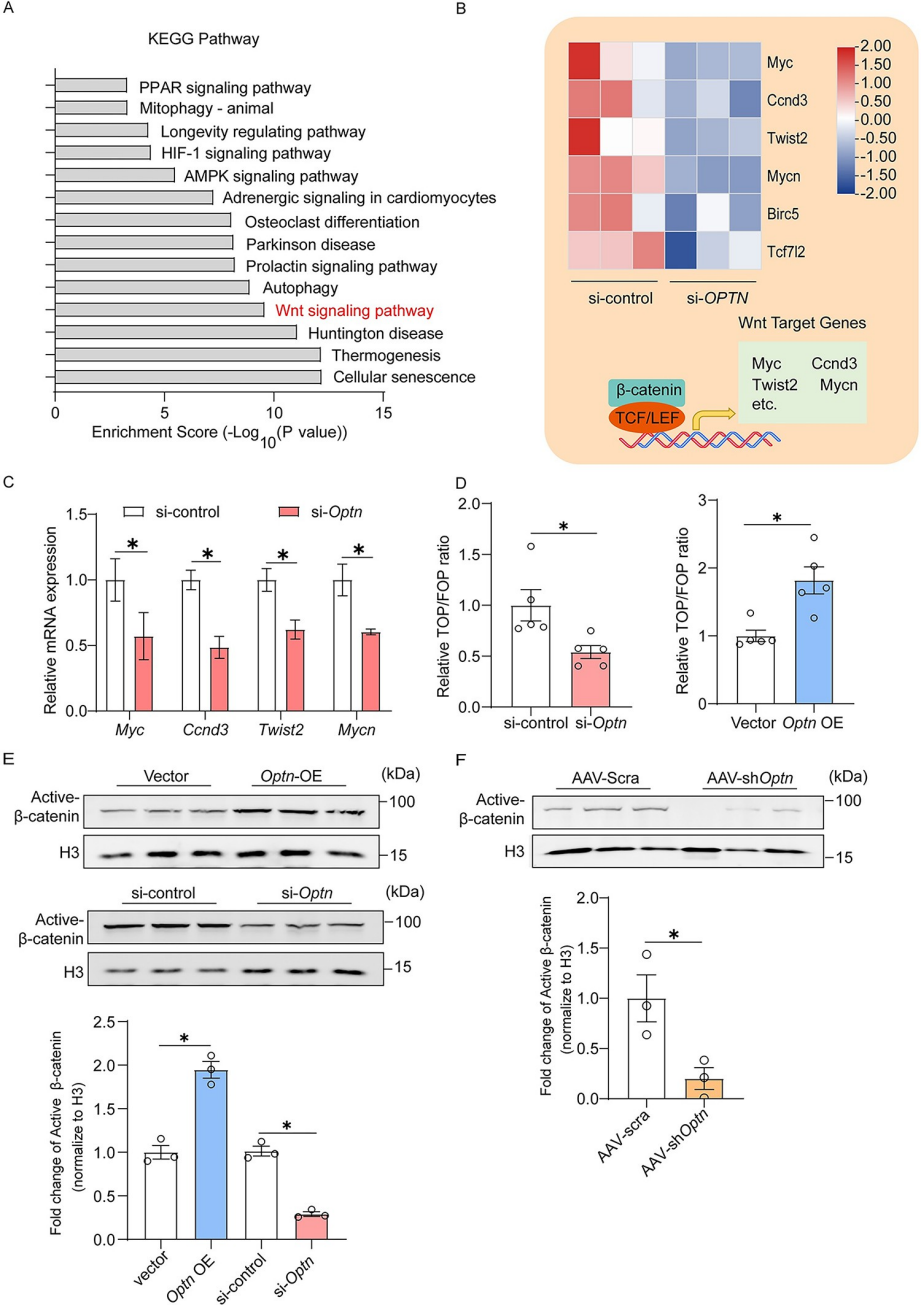
OPTN activates canonical Wnt signaling pathway in myoblasts. **(A)** KEGG pathway enrichment analysis in si-*Optn* Hela cells from the GEO dataset (GSE6819). **(B)** Heatmap of the changes in selected Wnts target genes expression levels in si-control and si-*OPTN* Hela cells by RNA-seq from the GEO dataset (GSE6819). **(C)** Representative mRNA expression analysis of Wnt target genes in si-control or si-*Optn* C2C12 cells at 4 days postdifferentiation (*n* = 3 in each group). **(D)** Representative luciferase activity of TOP/FOP in *Optn*-KD (left panel) and *Optn*-OE (right panel) C2C12 cells at 4 days postdifferentiation (*n* = 5 in each group). **(E, F)** Representative immunoblotting analysis and quantification of active β-catenin protein levels in nuclear lysates extracted from *Optn*-OE and *Optn* KD C2C12 cells at 4 days postdifferentiation (E) (*n* = 3 in each group) and in nuclear lysates extracted from scramble shRNA or sh*Optn* TA muscle at 5 days postinjury (F) (*n* = 3 mice in each group). Data are presented as mean ± SEM. *P < 0.05 versus control. The underlying data for this figure can be found in **S1 Data**. The original blot for this figure can be found in **S1 Raw Image**. AAV, adeno-associated viral vector; AMPK, AMP-activated protein kinase; Birc5, baculoviral IAP repeat containing 5; Ccnd3, cyclin D3; GEO, Gene Expression Omnibus; HIF, Hypoxia-inducible factor; H3, histone H3; KD, knockdown; KEGG, Kyoto Encyclopedia of Genes and Genomes; Myc, MYC proto-oncogene; Mycn, MYCN proto-oncogene; OE, overexpression; Optn, optineurin; PPAR, peroxisome proliferator-activated receptor; SEM, standard error of the mean; shRNA, short hairpin RNA; TA, tibialis anterior; Tcf7l2, transcription factor 7 like 2; TCF/LEF, T-cell factor/lymphoid enhancer factor; Twist2, twist family BHLH transcription factor 2.

**Fig 4:**
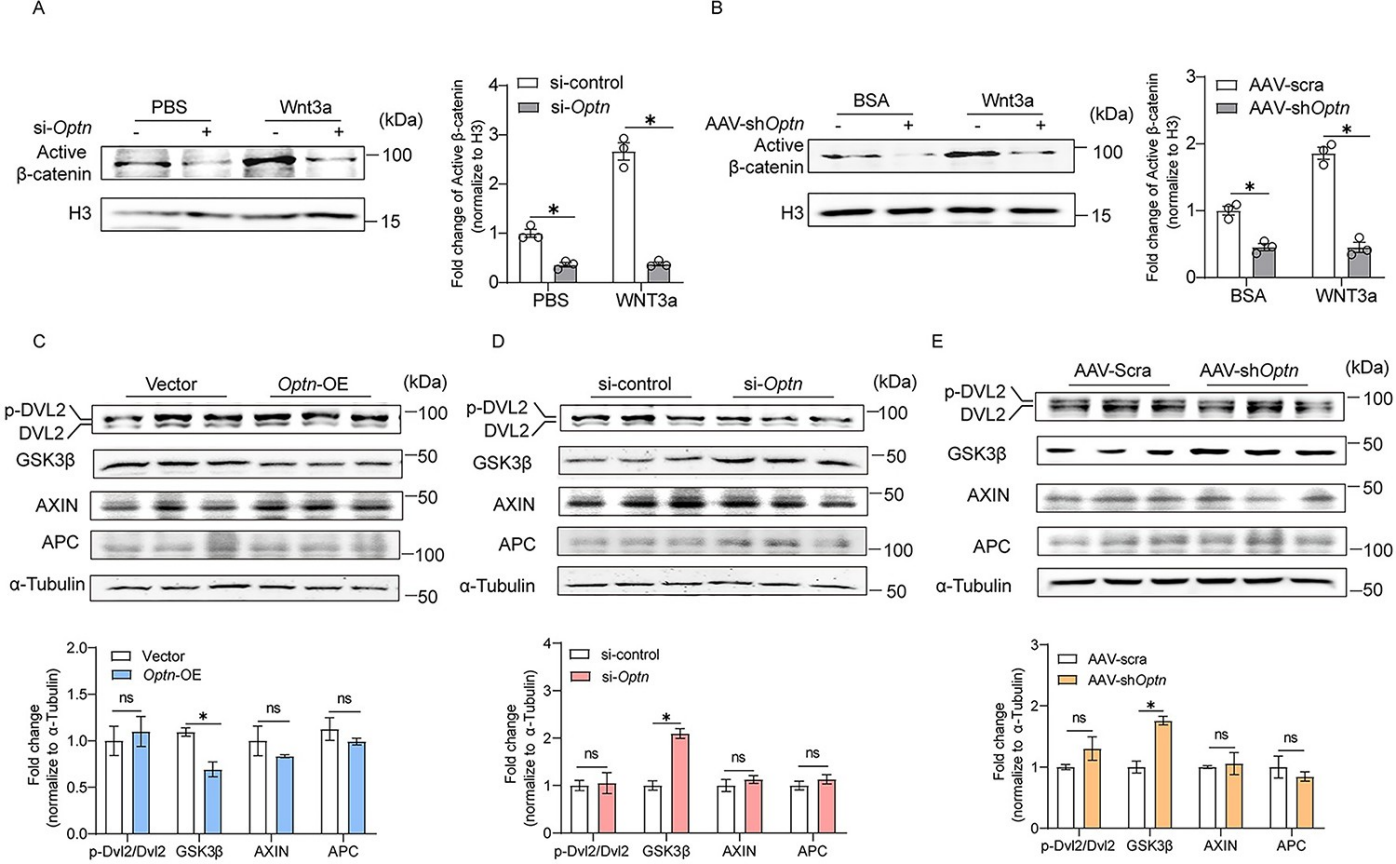
GSK3β is negatively regulated by OPTN during myogenesis. **(A, B)** Representative immunoblotting analysis and quantification of active β-catenin protein levels in nuclear lysates extracted from si-control or si-*Optn* C2C12 cells incubated with Wnt3a or PBS for 24 hours at 4 days postdifferentiation (A) (*n* = 3 independent experiment) and scramble shRNA or sh*Optn* TA muscle (intramuscular injection of Wnt3a at 1.5 days postinjury) at 5 days postinjury (B) (*n* = 3 mice in each group). **(C–E)** Representative immunoblotting analysis (upper panel) and quantification (lower panel) of DVL2 phosphorylation, GSK3β, AXIN, and APC protein levels in *Optn*-OE (C) and *Optn* KD (D) C2C12 cells at 4 days postdifferentiation (*n* = 3 in each group), as well as in scramble shRNA or sh*Optn* muscle TA muscle at 5 days postinjury (E) (*n* = 3 mice in each group). Data are presented as mean ± SEM. *P < 0.05 versus control. The underlying data for this figure can be found in **S1 Data**. The original blot for this figure can be found in **S1 Raw Image**. AAV, adeno-associated viral vector; APC, adenomatous polyposis coli protein; AXIN, axis inhibition protein; BSA, bovine serum albumin; DVL2, disheveled-2; GSK3β, glycogen synthase kinase 3β; H3, histone H3, KD, knockdown; OE, overexpressing; Optn, optineurin; SEM, standard error of the mean; shRNA, short hairpin RNA; TA, tibialis anterior.

To explore the underlying mechanism of OPTN-mediated myogenesis, we analyzed the available gene expression profiles in *Optn* KD Hela cells (GSE6819), a common model for deep transcriptome analysis [35,36] and a fast way to gain preliminary indications. The result showed high implication of OPTN in the regulation of Wnt signaling pathway (Fig 3A), a well-characterized pathway in myogenesis [15], with down-regulation of Wnt signaling pathway target genes [MYC proto-oncogene (*Myc*) [37], cyclin D3 (*Ccnd3*) [38], twist family BHLH transcription factor 2 (*Twist2*) [39], and MYCN proto-oncogene (*Mycn*) [40]] (Fig 3B). Consistent with this, we showed reduced mRNA levels of Wnts target genes in *Optn* KD C2C12 cells (Fig 3C). TCF/LEF is the major transcription factor of Wnts target genes [41]. The TOP/FOP reporter assay showed markedly decreased transcription activity of TCF/LEF in *Optn* KD C2C12 cells, while increased in *Optn*-OE C2C12 cells (Fig 3D). It has been shown that enhanced transcription activity of TCF/LEF is mediated by increased nuclear translocation of β-catenin, which further forms a complex with the TCF/LEF transcription factors to regulate Wnts target genes [17]. In agreement with this, *Optn* OE increased the nuclear levels of β-catenin in C2C12 cells, whereas *Optn* KD reduced these levels (Fig 3E, S7A and S7B Fig). Similarly, *Optn* KD also decreased nuclear levels of active β-catenin in TA muscle at day 5 postinjury in mice (Fig 3F). Furthermore, treatment of Wnt3a, a classical ligand of the Wnt signaling pathway [20], failed to effectively increase the nuclear levels of β-catenin in *Optn* KD C2C12 cells (Fig 4A) and TA muscle at day 5 postinjury (Fig 4B), suggesting that OPTN is required for the activation of canonical Wnt signaling pathway. Together, these results suggest that OPTN activates canonical Wnt signaling pathway during myogenesis.

### OPTN activates canonical Wnt signaling pathway through inhibition of GSK3β

To further investigate how OPTN promotes nuclear translocation of active β-catenin, we measured the phosphorylated and total levels of disheveled-2 (DVL2), a well-recognized positive regulator of the Wnt signaling pathway [42], and found no changes in *Optn* OE (Fig 4C) or KD C2C12 cells (Fig 4D), as well as in sh*Optn* muscle at day 5 postinjury (Fig 4E) when compared with their respective controls. The axis inhibition protein (AXIN)/adenomatous polyposis coli protein (APC)/GSK3β destruction complex is the negative regulator of the Wnt/β-catenin pathway [43–45]. *Optn* OE in C2C12 cells significantly reduced protein levels of GSK3β without affecting the protein levels of AXIN and APC (Fig 4C). In line with this, *Optn* KD in C2C12 cells (si-*Optn*) (Fig 4D) and skeletal muscle (sh*Optn)* at day 5 postinjury in mice (Fig 4E) increased GSK3β levels, with no changes in expression levels of AXIN and APC. Together, these results suggest that OPTN activates canonical Wnt signaling pathway through inhibition of GSK3β during myogenesis.

### OPTN physically interacts with and targets GSK3β for autophagic degradation

**Fig 5:**
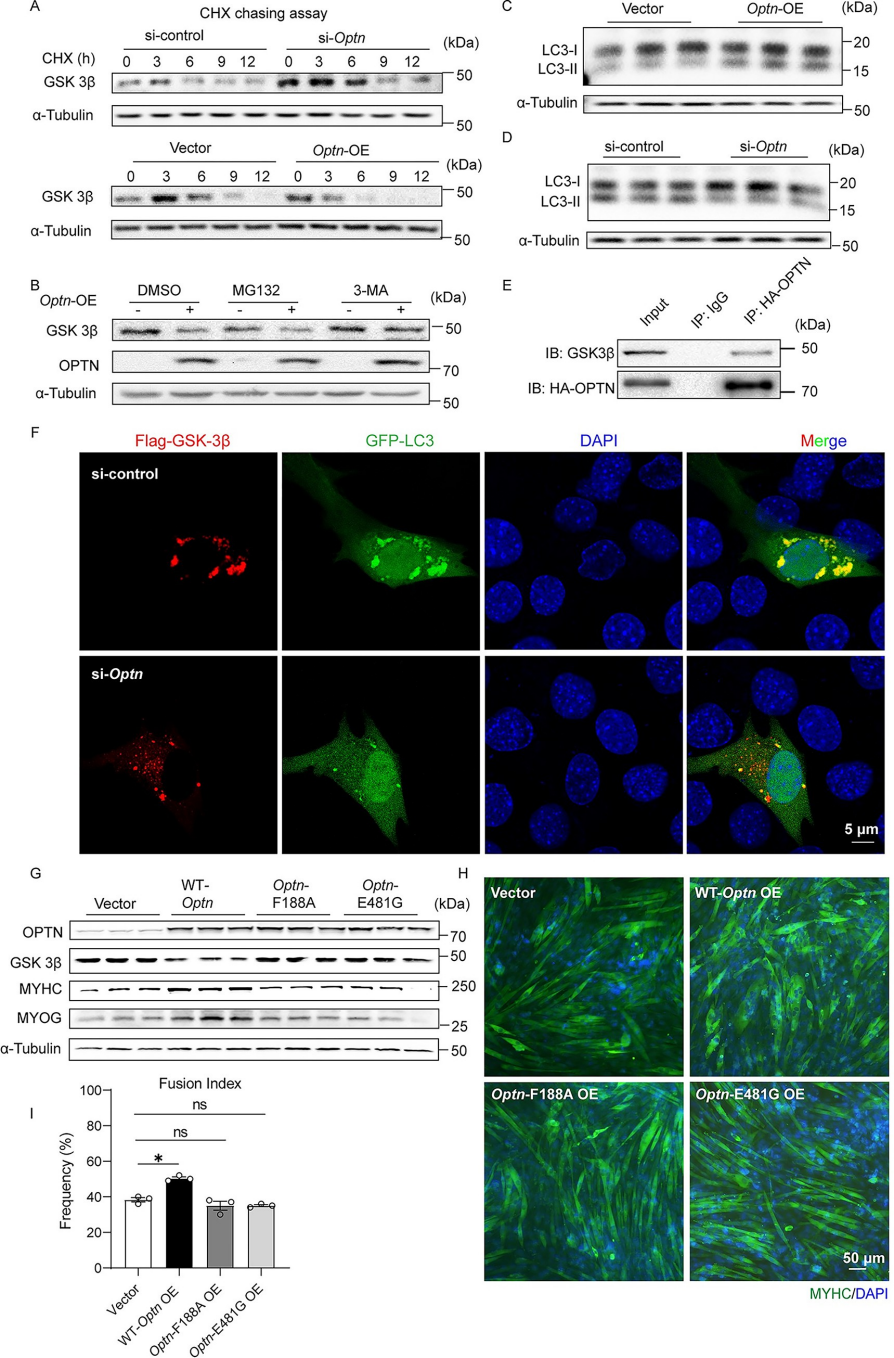
OPTN degrades GSK3β via activating LC3-mediated autophagy. **(A)** Representative immunoblotting analysis of GSK3β in *Optn* KD (up panel) and *Optn* OE (down panel) C2C12 cells at 4 days postdifferentiation and then treated with 50 μg/ml CHX at indicated time points (*n* = 3 in each group). **(B)** Representative immunoblotting analysis of GSK3β in control (empty vector) or *Optn*-OE C2C12 cells at 4 days postdifferentiation and then treated with DMSO, the proteasome inhibitor MG132 (25 μM) or the autophagy inhibitor 3-MA (5 mM) for 6 hours (*n* = 3 in each group). **(C, D)** Representative immunoblotting analysis of LC3 in *Optn* OE (C) and *Optn* KD (D) C2C12 cells at 4 days postdifferentiation (*n* = 3 in each group). **(E)** Immunoprecipitation of OPTN and endogenous GSK3β in Optn OE C2C12 cells. The immunoprecipitation analysis was performed in HA-Optn OE C2C12 cells incubated with anti-HA antibody or nonspecific Rabbit IgG (control) to pulldown endogenous GSK3β. **(F)** Representative immunofluorescence analysis of GFP-LC3 and FLAG-GSK3β in si-control and si-*Optn* C2C12 cells at 4 days postdifferentiation. Scale bars: 5 μm. **(G)** Representative immunoblotting analysis of OPTN, GSK3β, MYOG, and MYHC in empty vector, WT-*Optn*, *Optn*-F188A, and *Optn*-E481G OE C2C12 cells at 4 days postdifferentiation (*n* = 3 in each group). **(H, I)** Representative immunofluorescence staining of MYHC (H) and quantification of the fusion index (I) in vector, WT-*Optn*, *Optn*-F188A, and *Optn*-E481G OE C2C12 cells at 4 days postdifferentiation (*n* = 3 in each group). The plasmid for empty pcDNA 3.1-HA vector, pcDNA 3.1-HA-*Optn*, pcDNA 3.1-HA-*Optn*-F188A, and pcDNA 3.1-HA-*Optn*-E481G were transfected into C2C12 cells for 48 hours before the initiation of differentiation. Scale bars: 50 μm. Data are presented as mean ± SEM. *P < 0.05 versus control. The underlying data for this figure can be found in **S1 Data**. The original blot for this figure can be found in **S1 Raw Image**. CHX, cycloheximide; GSK3β, glycogen synthase kinase 3β; KD, knockdown; Optn, optineurin; OE, overexpressing; MYHC, myosin heavy chain; MYOG, myogenin; SEM, standard error of the mean; 3-MA, 3-methyladenine.

Given that OPTN is a selective autophagy receptor [11], we deduced that OPTN might directly mediate the degradation of GSK3β. Cycloheximide (CHX) chasing assay showed approximately 7 hours half-life of GSK3β in si-control but approximately 10 hours in *Optn* KD cells (Fig 5A, S8A Fig). In contrast, *Optn* OE decreased the half-life of GSK3β from approximately 8 hours to approximately 5 hours (Fig 5A, S8A Fig). These results indicate that OPTN accelerates the degradation of GSK3β. The OPTN-mediated degradation of GSK3β could be blocked by the autophagy inhibitor 3-methyladenine (3-MA), but not by the proteasome inhibitor MG132 (Fig 5B, S8B Fig). We further demonstrated that the reduction of GSK3β shown in *Optn* OE was abolished in autophagy deficient *ATG5* KO HEK 293T cells (S8C and S8D Fig). Meanwhile, the ratio of LC3II toI, which correlates with autophagic flux and represents a reliable index of autophagy [46], was up-regulated in *Optn* OE C2C12 cells, while down-regulated in *Optn* KD C2C12 cells (Fig 5C and 5D, S8E Fig). These results indicate that OPTN degrades GSK3β through autophagy pathway. Immunoprecipitation assay revealed protein–protein interaction between OPTN and GSK3β in C2C12 cells (Fig 5E). Immunofluorescence staining analysis showed colocalization of OPTN, GSK3β, and LC3 in C2C12 cells (S8F Fig). *Optn* KD reduced the interaction of LC3 and GSK3β in TA muscle at day 5 postinjury in mice as shown by immunoprecipitation (S8G Fig). Consistent with this, *Optn* KD decreased the colocalization of LC3 and GSK3β in C2C12 cells as shown by immunofluorescence staining (Fig 5F). Since OPTN has been shown to mediate autophagic degradation through LC3-interacting region (LIR) motif binding with LC3/GABARAP and UBANs binding with ubiquitin [47–49], we constructed the 2 mouse *Optn* point-mutants (F188A in LIR motif domain and E481G in UBAN domain) that are homologous with human mutants associated with its autophagic function [7,47,50] (S8H Fig). Compared with wild-type (WT) *Optn*, OE of either mutant in C2C12 cells failed to decrease the GSK3β levels (Fig 5G, S8I Fig), nor did it promote myoblast differentiation (Fig 5H and 5I), accompanied with reduced levels of MYOG and MYHC (Fig 5G, S8I Fig). These data indicate that OPTN degrades GSK3β via activating LC3-mediated autophagy.

### Inhibition of GSK3β rescues impaired myogenesis in *Optn* KD cells and skeletal muscle

**Fig 6:**
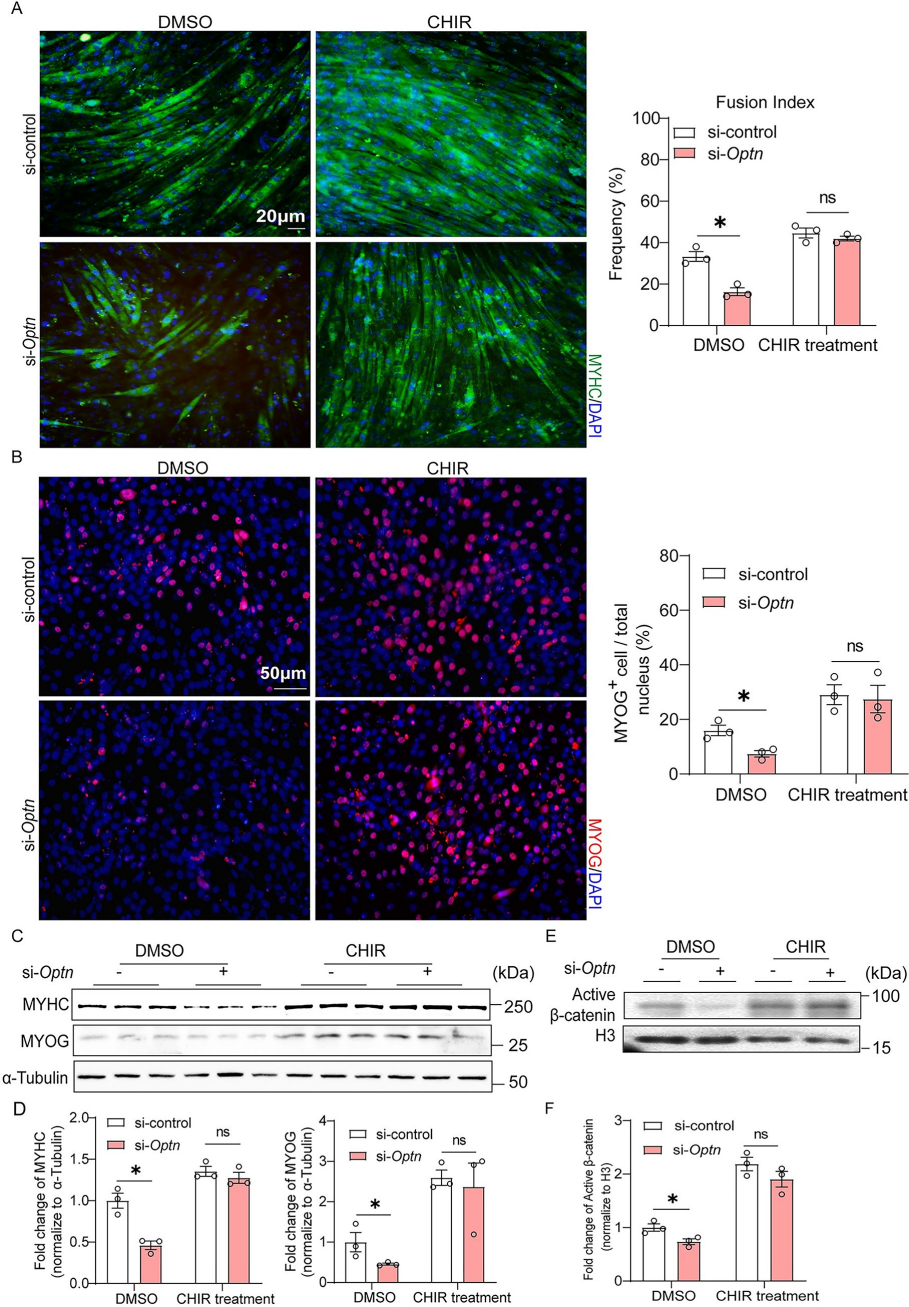
Inhibition of GSK3β rescues impaired myogenesis during myoblast differentiation induced by *Optn* KD. **(A)** Representative immunofluorescence staining of MYHC (left panel) and quantification of fusion index (a MYHC^+^ cell with at least 3 nucleus) (right panel) in control (si-control) and *Optn* KD (si-*Optn*) C2C12 cells treated with CHIR (20 μM) or DMSO at 4 days postdifferentiation (*n* = 3 in each group). si-control or si-*Optn* were transfected into C2C12 cells for 48 hours before the initiation of differentiation. Scale bars: 20 μm. **(B)** Representative immunofluorescence staining of MYOG (left panel) and the percentage of MYOG^+^ cells (right panel) in control (si-control) and *Optn* KD (si-*Optn*) C2C12 cells treated with CHIR or DMSO at 4 days postdifferentiation (*n* = 3 in each group). si-control or si-*Optn* were transfected into C2C12 cells for 48 hours before the initiation of differentiation. Scale bars: 50 μm. **(C, D)** Representative immunoblotting analysis (C) and quantification (D) of MYOG and MYHC in control (si-control) and *Optn* KD (si-*Optn*) C2C12 cells treated with CHIR or DMSO at 4 days postdifferentiation (*n* = 3 in each group). si-control or si-*Optn* were transfected into C2C12 cells for 48 hours before the initiation of differentiation. **(E, F)** Representative immunoblotting analysis (E) and quantification (F) of active β-catenin protein levels in nuclear lysates extracted from si-control and si-*Optn* C2C12 cells treated with CHIR or DMSO at 4 days postdifferentiation (*n* = 3 in each group). si-control or si-*Optn* were transfected into C2C12 cells for 48 hours before the initiation of differentiation. Data are presented as mean ± SEM. *P < 0.05 versus control. The underlying data for this figure can be found in **S1 Data**. The original blot for this figure can be found in **S1 Raw Image**. GSK3β, glycogen synthase kinase 3β; H3, histone H3; KD, knockdown; Optn, optineurin; MYHC, myosin heavy chain; MYOG, myogenin; SEM, standard error of the mean.

**Fig 7:**
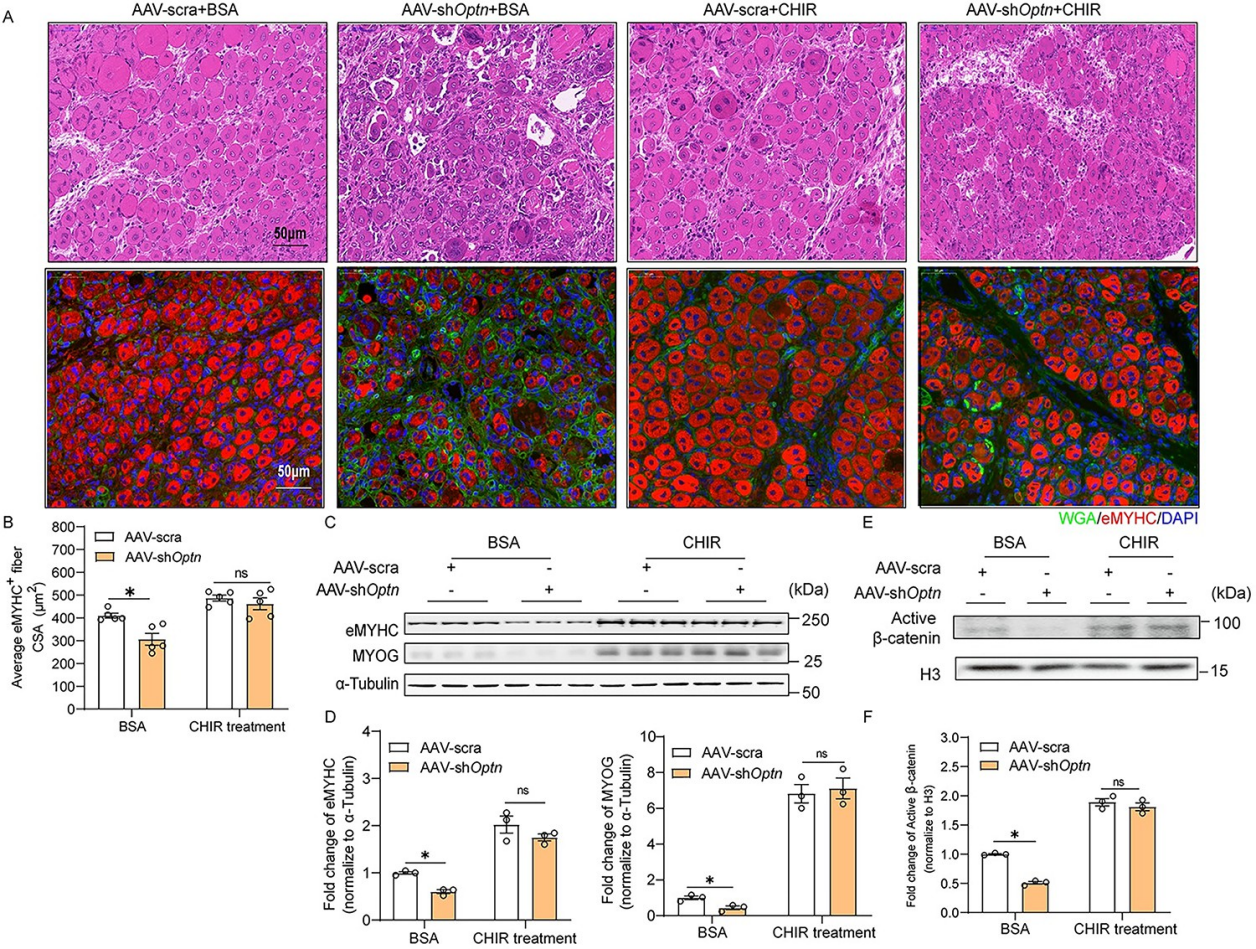
Inhibition of GSK3β rescues impaired skeletal muscle regeneration induced by *Optn* KD. **(A)** Representative HE staining and immunofluorescence analysis of eMYHC^+^ fibers in scramble shRNA or sh*Optn* TA muscle (intramuscular injection of CHIR or BSA at 2.5 days postinjury) at 5 days postinjury (*n* = 5 mice in each group). Scale bar = 50 μm. **(B)** Average CSA of regenerating eMYHC^+^ myofibers at 5 days postinjury (*n* = 5 mice in each group). **(C, D)** Representative immunoblotting analysis (C) and quantification (D) of myogenic markers (eMYHC and MYOG) in scramble shRNA or sh*Optn* TA muscle (intramuscular injection of CHIR or BSA at 2.5 days postinjury) at 5 days postinjury (*n* = 3 mice in each group). **(E, F)** Representative immunoblotting analysis (E) and quantification (F) of active β-catenin protein levels in nuclear lysates extracted from scramble shRNA or sh*Optn* TA muscle (intramuscular injection of CHIR or BSA at 2.5 days postinjury) at 5 days postinjury (*n* = 3 mice in each group). Data are presented as mean ± SEM. *P < 0.05 versus control. The underlying data for this figure can be found in **S1 Data**. The original blot for this figure can be found in **S1 Raw Image**. AAV, adeno-associated viral vector; BSA, bovine serum albumin; CSA, cross-sectional fiber area; eMYHC, embryonic myosin heavy chain; GSK3β, glycogen synthase kinase 3β; HE, hematoxylin–eosin; H3, histone H3; Optn, optineurin; SEM, standard error of the mean; shRNA, short hairpin RNA; WGA, wheat germ agglutinin; TA, tibialis anterior.

To determine whether GSK3β is required for OPTN-mediated myogenesis, we inhibited the activity of GSK3β by its specific inhibitor CHIR and found rescued cell fusion and multinuclear myotube formation events in *Optn* KD cells (Fig 6A). The numbers of MyoG^+^ cells were restored in *Optn* KD C2C12 cells treated with CHIR (Fig 6B). Consistent with the morphological improvement, CHIR treatment recovered the down-regulated expression levels of MYHC and MYOG in *Optn* KD differentiating myoblasts (Fig 6C and 6D) and rescued the reduced nuclear levels of β-catenin in *Optn* KD C2C12 cells (Fig 6E and 6F). When CHIR was injected into the TA muscle treated with AAV-sh*Optn* after CTX injury, the small size of eMYHC^+^ regenerating fibers at day 5 postinjury was efficiently rescued (Fig 7A and 7B), with concurrent restoration of eMYHC, MYOG and nuclear β-catenin levels (Fig 7C–7F). Cumulatively, these data suggest that OPTN activates canonical Wnt signaling mediated myogenesis during muscle regeneration through degradation of GSK3β.

## Discussion

The regeneration ability of skeletal muscle has a high clinical relevance in some conditions such as postinjury recovery and muscular disease, and myogenic differentiation–mediated myogenesis is a pivotal step of muscle regeneration [2,51]. Here, we identified a selective autophagy receptor OPTN from 5 independent microarray datasets as a potential regulator of myogenesis. By performing a series of in vivo and in vitro experiments, we for the first time showed that OPTN activates Wnt signaling pathway mediated myogenesis through autophagic degradation of GSK3β during muscle regeneration. Our findings extend the understanding of regulatory mechanisms upon Wnt signaling pathway during muscle regeneration and reveal OPTN as a potential therapeutic target for defective muscle regeneration.

OPTN promotes myogenesis via activating canonical Wnt signaling pathway. The pathway is critical for myogenesis during muscle regeneration [52], regulating SCs differentiation through β-catenin binding with transcription factor 4 (TCF4) on the promoter of MYOG [53,54]. The stabilization of β-catenin is regulated by its destruction complex components. GSK3β, an important component of β-catenin destruction complex [45], is inhibited by Wnts to stabilize β-catenin and activate its nuclear translocation. There are several interpretations on Wnt-mediated inhibition of GSK3β in different models, including dissociation of GSK3β from AXIN via conformational changes [55] or posttranslational modifications [56], recruitment of GSK3 inhibitory proteins such as frequently rearranged in advanced T-cell lymphomas (FRATs) [57], or degradation of AXIN [58]. In addition, Wnts were reported to inhibit GSK3β activity by promoting its sequestration from the cytosol into multivesicular endosomes/bodies fusing with lysosome in 293T cells [59,60]. These findings shed fresh light on the inhibition of GSK3β and arouse broad interest in the field [61]. Based on the fact that the multivesicular endosome/body is an obligatory step before degradation in autophagosome [62,63], degradation might be the final fate of Wnt-induced GSK3β sequestration. Consistent with this, our data show GSK3β degradation mediated by OPTN in autophagosome during myogenesis, corroborating that OPTN involves in Wnt-mediated inhibition of GSK3β. These findings expand insights into the canonical Wnt/GSK3β/β-catenin signaling and reveal a novel mechanism on GSK3β inhibition in skeletal muscle.

OPTN is an autophagy receptor that plays a central role in selective autophagy [9]. Our data demonstrate that OPTN activates Wnt signaling pathway through autophagic degradation of GSK3β in muscle, suggesting an interaction between autophagy and Wnt signaling pathway during myogenesis. In fact, conflicting results on their relationship have been reported. Up-regulation of autophagy is observed during the formation of mature myotubes and muscle regeneration [64–66], whereas inhibition of DVL2 autophagic degradation (a positive regulator of Wnt signaling pathway) is paradoxically capable of promoting Wnt signaling during muscle regeneration [67]. Similar results have been confirmed by Gao and colleagues, showing that autophagy inhibits Wnt signaling pathway by promoting Dvl2 degradation [68]. These results suggest negative regulation of Wnt signaling pathway by autophagy. Nevertheless, it has recently been reported that autophagy directly promotes nuclear translocation of β-catenin in a GSK3β dependent manner in C2C12 cells [69]. Consistently, our findings suggested that autophagy mediated OPTN-GSK3β axis exerts positive effects on Wnt signaling pathway during myogenic differentiation upon injury. In line with this, during hepatic progenitor cell differentiation, autophagy activates Wnt signaling pathway through interaction of p62 and phosphorylated GSK3β [70]. During adipocyte differentiation, a positive regulator of autophagy tumor protein P53 inducible nuclear protein 2 (TP53INP2) activates Wnt signaling pathway through autophagy-dependent sequestration of GSK3β [61]. Together, these findings suggest that the indeterminate intercommunication between autophagy and Wnt signaling pathway might be affected by dissimilar (negative or positive) regulators.

**Fig 8:**
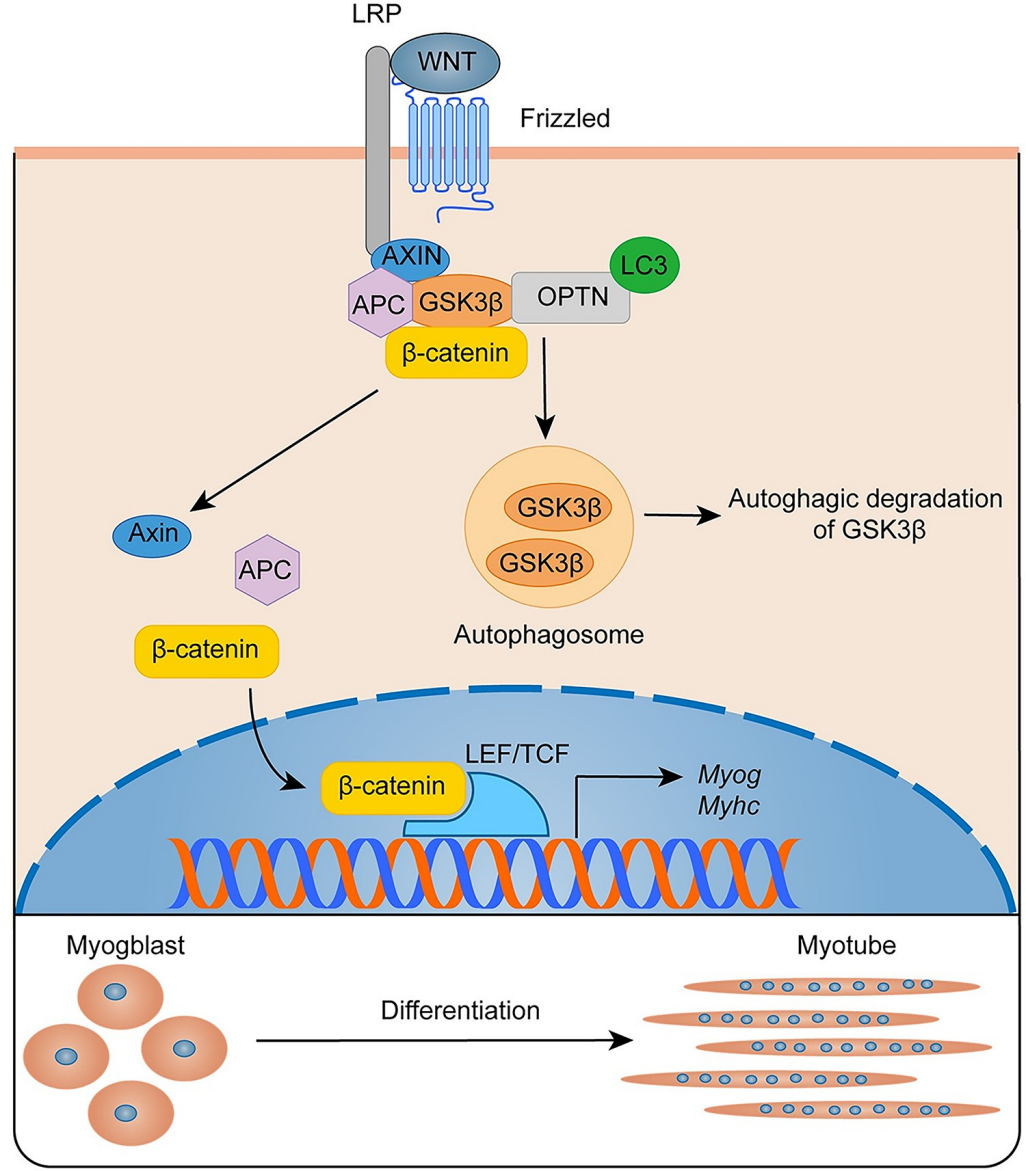
Schematic model of the role of OPTN on myogenesis during muscle regeneration. OPTN physically interacts with and targets GSK3β for autophagic degradation and then promotes the nuclear translocation of β-catenin, resulting in activation of Wnt signaling pathway mediated myogenesis during muscle regeneration. APC, adenomatous polyposis coli protein; AXIN, axis inhibition protein; GSK3β, glycogen synthase kinase 3β; LEF/TCF, lymphoid enhancer factor/T-cell factor; LRP, low-density lipoprotein receptor related protein, OPTN, optineurin.

In summary, our data identify a novel function of OPTN for myogenesis during muscle regeneration. OPTN promotes myogenesis during muscle regeneration through autophagic degradation of Wnt signaling pathway inhibitor GSK3β (Fig 8). These findings uncover an OPTN/GSK3β/β-catenin axis that regulates myogenesis during muscle regeneration. Thus, OPTN may be a potential therapeutic target for the prevention and treatment of impaired myogenesis in injury and other muscular disease, such as DMD [71] and aging [72]. Further investigation is needed to assess the myogenic function of OPTN in advanced models to increase its impact on translational medicine.

## Materials and methods

### Animal studies

Six-week-old male C57BL/6J mice purchased from the animal center of Xi’An Jiao Tong University (Xi’an, Shaanxi, PRC) were performed in accordance with the National Institutes of Health (Bethesda, Maryland, United States of America) Guide for the Care and Use of Laboratory Animals and with the approval of Animal Ethical and Welfare Committee of Northwest A&F University (Yang Ling, Shaanxi, PRC) [Approval ID: NWAFU-314031143]. All mice were housed with a 12-hour dark/light cycle with food and water ad libitum and were randomly allocated to the indicated groups. AAV serotype 9 vectors encoding a control scrambled shRNA sequence (scrambled; 5′-TTCTCCGAACGTGTCACGTAA-3′) or a short hairpin targeting OPTN (sh*Optn*; 5′-GCAAATGGCCATTCTTCTA-3′) under the control of a U6 promoter and expressing EGFP (driven by a CMV promoter) were obtained from Hanbio (Shanghai, PRC). A single dose of 1.1 × 10^12^ vg/mice in 40 μL of AAV2/9 expressing sh*Optn* was delivered to 8-week-old mice injected locally into the right TA muscle, and the same dose of AAV2/9 expressing shRNA control was injected left TA muscle as AAV-shRNA control group. Production and purification of recombinant AAV were made by Hanbio. Mice were treated after recombinant AAV injection for 4 weeks. To induce muscle injury, 50 μl of 10 μM CTX (HF005, Heng Fei Biotechnology, Shanghai, PRC) was injected into TA muscle. The TA muscle was harvested at 0, 3, 5, and 14 days postinjury. To verify the activity of canonical Wnt signaling in vivo, the TA muscle of scrambled shRNA or sh*Optn* was injected with 20 μl Wnt3a (100 ng/ml, 315–20, PeproTech, New Jersey, USA) or PBS (0.1% BSA) per mouse at 1.5 days postinjury [67]. The injured TA muscle was then collected for western blot analysis at 5 days postinjury. To inhibit the activity of GSK3β in vivo, the TA muscle of scrambled shRNA or sh*Optn* was injected with 20 μl CHIR-99021 (50 ng/ml, HY-13259, MedChemExpress, Shanghai, PRC) or PBS (0.1% BSA) per mouse at 2.5 days postinjury [67]. The injured TA muscle was then collected for hematoxylin–eosin (HE) analysis and western blotting at 5 days postinjury.

### Histological analysis

The TA muscle was fixed with 4% paraformaldehyde for more than 72 hours and then subjected to dehydration embedding. Finally, paraffin sections of muscle were obtained at a thickness of 2 to 4 μm for HE staining, and whole-slide digital images were collected with an Pannoramic DESK Scanner (P-MIDI, P250, 3D HISTECH, Hungary).

### 5-Ethynyl-2′-deoxyuridine assays in vivo and in vitro

Mice were given intraperitoneal injection of 5-Ethynyl-2′-deoxyuridine (EdU) (50 mg/kg body weight, intraperitoneal injection; HY-118411, MedChemExpress) 2 consecutive days before analyzed. EdU was detected with the Cell-Light Apollo 567 Stain Kit (C10317-1, RiboBio, Guangzhou, PRC). About 200 nucleus per sample from one mouse were counted. EdU and Pax7 double-positive nucleus, EdU-positive nucleus, and total cell nucleus were counted using ImageJ. The proliferation of C2C12 myoblasts cultured was determined using the Cell-Light EdU Apollo 567 In Vitro Kit (C10310-1, RiboBio) according to manufacturer’s instructions. EdU-positive nucleus and total cell nucleus were counted using ImageJ.

### Cell culture

C2C12 cells were purchased from China Infrastructure of Cell Line Resource and were cultured in growth medium comprising high-glucose Dulbecco’s Modified Eagle medium (DMEM) (H30022.01, HyClone, Connecticut, USA) supplemented with 10% fetal bovine serum (FBS) (Z7186FBS-500, ZETA LIFE, California, USA), 1% penicillin/streptomycin. After 48 hours, C2C12 cells were cultured in differentiation medium (high-glucose DMEM supplemented with 2% horse serum and 1% penicillin/streptomycin). Atg5^+/+^ and Atg5^−/−^ HEK293T cell lines were maintained in DMEM supplemented with 10% FBS and 1% penicillin/streptomycin. Atg5^+/+^ and Atg5^−/−^ HEK293T cell lines were kindly provided by Dr. Jun Cui (School of Life Sciences, Sun Yat-sen university).

### Plasmids and RNA interference

In order to construct a plasmid encoding HA-OPTN, HA-OPTN-F188A, and OPTN-E481G, the HA-tag was added at the N-terminus of OPTN, OPTN-F188A, and OPTN-E481G. The WT-OPTN, OPTN-F188A, and OPTN-E481G were cloned into the BamHI and XhoI sites of the pcDNA3.1-HA vector. In order to construct a plasmid encoding FLAG-GSK3β, the FLAG tag was added at the N-terminus of GSK3β. GSK3β was then cloned into the NotI and XbaI sites of the pcDNA3.1-FLAG vector. The pcDNA3.1-HA, pcDNA3.1-FLAG, and GFP-LC3B plasmid were provided by Dr. Qingzhu Sun (College of Animal Science and Technology, Northwest A&F University). The TOP flash/FOP flash and Renilla luciferase expression plasmids was provided by Dr. Qingyong Meng (College of Biological Sciences, China Agricultural University). The si-control, si-*Optn*, and si-β-catenin were synthesized from GenePharma (Shanghai, PRC). The sequences of *Optn* and β-catenin siRNAs were as follows: *Optn* siRNA1, 5′-GCAGACUUACCUGUUUCAATT-3′; *Optn* siRNA2, 5′-GCAAAUGGCCAUUCUUCUATT-3′.

### Plasmid transfection and luciferase reporter assay

The plasmids were transfected into C2C12 cells and HEK293T cells using Lipofectamine 3000 (L3000001, Invitrogen, California, USA). In order to identify the activity of canonical Wnt signaling, the TOP flash/FOP flash expression plasmids with the Renilla luciferase expression plasmid were transfected when si-control and si-*Optn* C2C12 cells or HA-vector and HA-Optn were cultured after 2 days in the differentiation medium. The reporter activity was measured using the Dual-luciferase Reporter Assay System (E1980, Promega, Wisconsin, USA).

### Immunofluorescence

Muscle sections and cultured cells were fixed in 4% formaldehyde for 10 minutes, permeabilized with 0.2% Triton X-100 for 20 minutes on ice, and then blocked in 3% bovine serum albumin in PBS for 1 hour. The samples were blocked in 5% BSA for 2 hours at room temperature. Primary antibodies listed in S1 Table were incubated in blocking buffer at 4°C overnight. Subsequently, the samples were washed with PBS and stained with the appropriate fluorescently labeled secondary antibodies (fluorescein isothiocyanate or rhodamine) for 1 hour at room temperature. After washing with PBS, DAPI (C0060, Solarbio, Beijing, PRC) was used to stain nucleus for 3 minutes. For immunostaining of muscle sections, whole-slide digital images were collected with an Pannoramic DESK Scanner (P-MIDI, P250, 3D HISTECH). Cross-sectional area of the new myofibers was calculated on section images obtained from TA muscle using ImageJ. For immunostaining of cultured cells, images were acquired using BioTEK gen 5 Software. Total cell nucleus and nucleus within myotubes were counted using ImageJ. The distribution of nucleuses per myotube and fusion index (a MYHC^+^ cell with at least 3 nucleuses) was calculated as the number of nucleuses in myotubes divided by the total number of nucleuses counted.

### Real-time reverse transcriptase PCR

Real-time PCR were performed as described [73]. Total RNA was isolated from the fresh TA muscle using TRIzol reagent (9109, Takara, Shiga, Japan). Complementary DNA (cDNA) was synthesized from total RNA using cDNA synthesis kit (R333-01, Vazyme Biotech, Nanjing, China) following the manufacturer’s instructions. RT-PCR was performed using a CFX 96 Real-Time PCR Detection System (Bio-Rad, Hercules, California, USA). Each 20 mL amplifications contained 10 μl of ChamQ SYBR qPCR Master Mix (Q222-01, Vazyme Biotech), 7.8 mL of sterilized double-distilled water, 1 mL of 1:10 diluted cDNA, and 0.6 mL of each forward and reverse primer. The RT-qPCR program comprised an initial activation step at 95°C for 3 minutes, followed by 38 cycles of 95°C for 15 seconds and 60°C for 30 seconds, and 5 seconds at 65°C. After the PCR, a single product generated in these reactions was confirmed via melting curve analyses. The comparative Ct method (2^−ΔΔCt^), described in the literature [74] was used to calculate the gene expression values. The primer sequences for genes were listed in S2 Table.

### Immunoblotting

C2C12 cells and TA muscle were washed with PBS and lysed in RIPA lysis buffer (P0013C, Beyotime Biotechnology, Shanghai, PRC). Next, 200 μg of total protein was resolved by 10% or 12% sodium dodecyl sulfate-polyacrylamide gel electrophoresis (SDS-PAGE) electrophoresis and transferred onto a polyvinylidene fluoride (PVDF) (IPVH00010, Millipore, Massachusetts, USA) membrane via electroblotting. The PVDF membrane was blocked in black buffer (5% skim milk powder dissolved in TBST) for 2 hours at room temperature. Primary antibodies listed in S1 Table were applied in TBST at 4°C overnight. Subsequently, the PVDF membrane was washed 4 times with TBST (5 minutes per time) and stained with the secondary antibodies (goat anti-rabbit or mouse) for 2 hours at room temperature. After washing with TBST, the ECL Reagent (WBKlS0100, Millipore) was used, and the strips were on film.

### Immunoprecipitation

For immunoprecipitation analysis, the TA muscle and cultured cells were homogenized with IP lysis buffer (containing 1M pH 7.4 Tris-HCl 25ml, NP40 25ml, NaCl 4.383g, EDTA 0.146g, glycerin 50 ml, and protease inhibitor cocktail), and the total protein was incubated with 5 μg of the Rabbit monoclonal antibody to HA, GSK3β, or nonspecific Rabbit IgG for 2 hours at room temperature and then immunoprecipitation with protein A/G magnetic beads (B23201, Bimaker, Shanghai, PRC) at 4°C overnight. After washing 3 times with TPBS (5 minutes per time), the protein-bound beads were finally resuspended in 20 μl 1× SDS-PAGE loading buffer. The samples were boiled at 95°C for 10 minutes, and the supernatant was loaded on the gel for immunoblotting.

### Treatment with reagents in cell culture

In order to verify the activity of canonical Wnt signaling, si-control and si-OPTN C2C12 cells were treated with either Wnt3a (100 ng/ml, 315–20, PeproTech) or PBS for 24 hours after 2 days in differentiation medium. The cell samples were then collected for western blot analysis. In order to inhibit the activity of GSK3β in OPTN KD C2C12 cells, the OPTN KD C2C12 cells were treated with CHIR-99021 (CHIR; 3 μM, HY-10182, MedChemExpress) during differentiation. Western blotting and immunofluorescence analysis were performed after 4 days of differentiation. For CHX chasing assay, cells were treated with CHX (50 μg/ml) and collected at the indicated time points and prepared for western blot analysis. To determine which degradation system dominantly controls the degradation of GSK3β, DMSO, MG132 (25 μM, HY-13259, MedChemExpress), and 3-MA (5 mM, HY-19312, MedChemExpress) were added to C2C12 cells cultured for 6 hours with or without *Optn* OE to detect the protein expression of GSK3β via western blotting.

### Statistical analysis

All experiments were at least performed in 3 independent experiments. Data are presented as mean ± standard error of the mean and were analyzed by 2-tailed Student *t* tests for comparisons between 2 groups or 2-way analysis of variance (ANOVA) with Duncan post hoc test for multiple comparisons. Statistical significance was defined as *P < 0.05 versus controls. All data were analyzed using PASW Statistics 20 (SPSS, Chicago, Illinois, USA).

## Supporting information

S1 Fig The quantification of OPTN and eMYHC immunoblotting analysis during muscle regeneration. **(A)** The quantification of OPTN immunoblotting analysis in TA of WT mice at 0, 3, 5, and 14 days postinjury (*n* = 3 mice in each group). **(B)** The quantification of eMYHC immunoblotting analysis in TA of WT mice at 0, 3, 5, and 14 days postinjury (*n* = 3 mice in each group). Data are presented as mean ± SEM. *P < 0.05 versus control. The underlying data for this figure can be found in **S1 Data**. CTX, cardiotoxin; eMYHC, embryonic myosin heavy chain; OPTN, optineurin; SEM, standard error of the mean; TA, tibialis anterior; WT, wild-type.
(TIF)

S2 Fig Representative immunofluorescence analysis of OPTN and eMYHC in TA muscle at 5 days postinjury. The OPTN, newly regenerated myofibers, and nucleus were stained with anti-OPTN antibody (red), anti-eMYHC antibody (green), and DAPI (blue), respectively. Scale bars: 50 um. eMYHC, embryonic myosin heavy chain; OPTN, optineurin; TA, tibialis anterior.
(TIF)

S3 Fig The efficiency of OPTN KD in mouse TA muscle by AAV shRNA. **(A)** Representative immunoblotting analysis (left panel) and quantification (right panel) of the OPTN in C2C12 myoblasts with si-control or si-*Optn* #1–2 transfection (*n* = 3 in each group). **(B)** Representative fluorescence image at 4 weeks postinjection of AAV containing scramble RNA or sh*Optn*. **(C)** Quantification of *Optn* mRNA expression in TA muscle at 4 weeks postinjection of AAV containing scramble RNA or sh*Optn* (*n* = 5 mice in each group). Representative immunoblotting analysis (left panel) and quantification (right panel) of the OPTN in TA muscle at 4 weeks postinjection of AAV containing scramble RNA or sh*Optn* (*n* = 3 mice in each group). Data are presented as mean ± SEM. *P < 0.05 versus control. The underlying data for this figure can be found in **S1 Data**. The original blot for this figure can be found in **S1 Raw Image**. AAV, adeno-associated viral vector; KD, knockdown; OPTN, optineurin; SEM, standard error of the mean; shRNA, short hairpin RNA; TA, tibialis anterior.
(TIF)

S4 Fig OPTN does not affect cell proliferation. **(A)** Representative immunofluorescence staining of Pax7 (green), EdU (red), and DAPI (blue) in scramble shRNA or sh*Optn* TA muscle at 3 days postinjury. Scale bar: 50 μm. **(B)** Quantification of the percentage of Pax7^+^EdU^+^ cells in scramble shRNA or sh*Optn* TA muscle at 3 days postinjury (*n* = 5 mice in each group). **(C)** Representative immunofluorescence staining of Pax7 (red) and DAPI (blue) in scramble shRNA or sh*Optn* TA muscle at 3 days postinjury. Scale bar: 50 μm. **(D)** Quantification of the percentage of Pax7^+^ cells in scramble shRNA or sh*Optn* TA muscle at 3 days postinjury (*n* = 5 mice in each group). **(E)** Representative EdU and DAPI staining analysis in control (si-control) and *Optn* KD (si-*Optn*) C2C12 cells. si-control or si-*Optn* were transfected into C2C12 cells for 24 hours before staining analysis. Scale bar: 300 μm. **(F)** Quantification of the percentage of EdU-positive cells/total cells in control (si-control) and *Optn* KD (si-*Optn*) C2C12 cells (*n* = 5 in each group). si-control or si-*Optn* were transfected into C2C12 cells for 24 hours before staining analysis. **(G)** Representative mRNA expression analysis of cell proliferation–associated genes in C2C12 cells with si-control or si-*Optn* transfection (*n* = 6 in each group). Cells were collected after 24h transfection. Data are presented as mean ± SEM. *P < 0.05 versus control. The underlying data for this figure can be found in **S1 Data**. AAV, adeno-associated viral vector; EdU, 5-Ethynyl-2′-deoxyuridine; KD, knockdown; OPTN, optineurin; Pax7, paired box 7; SEM, standard error of the mean; shRNA, short hairpin RNA; TA, tibialis anterior.
(TIF)

S5 Fig The quantification of OPTN and MYOG immunoblotting analysis in C2C12 cells during differentiation. **(A)** The quantification of OPTN immunoblotting analysis in C2C12 cells during differentiation at the indicated time points (0, 2, 4, 6, and 8 days) (*n* = 3 in each group). **(B)** The quantification of MYOG immunoblotting analysis in C2C12 cells during differentiation at the indicated time points (0, 2, 4, 6, and 8 days) (*n* = 3 in each group). Data are presented as mean ± SEM. *P < 0.05 versus control. The underlying data for this figure can be found in **S1 Data**. MYOG, myogenin; Optn, optineurin; SEM, standard error of the mean.
(TIF)

S6 Fig OPTN promotes myoblast differentiation mediated myogenesis. **(A, B)** Representative immunoblotting analysis (A) and quantification (B) of OPTN, MYHC, and MYOG in si-control or si-*Optn* C2C12 cells at 4 days postdifferentiation (*n* = 3 in each group). si-control or si-*Optn* were transfected into C2C12 cells for 48 hours before the initiation of differentiation. **(C, D)** Representative immunoblotting analysis (C) and quantification (D) of OPTN, MYHC and MYOG in control (empty vector) and *Optn* OE C2C12 cells at 4 days postdifferentiation (*n* = 3 in each group). The empty pcDNA 3.1-HA vector or pcDNA 3.1-HA-*Optn* vector were transfected into C2C12 cells for 48 hours before the initiation of differentiation. Cells were collected at 4 days postdifferentiation. Data are presented as mean ± SEM. *P < 0.05 versus control. The underlying data for this figure can be found in **S1 Data**. The original blot for this figure can be found in **S1 Raw Image**. MYOG, myogenin; MYHC, myosin heavy chain; OE, overexpressing; Optn, optineurin; SEM, standard error of the mean.
(TIF)

S7 Fig OPTN enhances nuclear β-catenin levels in C2C12 cells. **(A, B)** Representative immunofluorescence analysis of active β-catenin in *Optn* OE (A) and *Optn* KD (B) C2C12 cells at 4 days postdifferentiation. Scale bar: 10 μm. KD, knockdown; OE, overexpressing; Optn, optineurin.
(TIF)

S8 Fig OPTN degrades GSK3β via activating LC3-mediated autophagy. **(A)** The quantification of GSK3β immunoblotting analysis in *Optn* KD (left panel) and *Optn* OE (right panel) C2C12 cells at 4 days postdifferentiation and then treated with 50 μg/ml CHX at indicated time points (*n* = 3 in each group). **(B)** The quantification of GSK3β immunoblotting analysis in control (empty vector) or *Optn*-OE C2C12 cells at 4 days postdifferentiation and then treated with DMSO, the proteasome inhibitor MG132 (25 μM), or the autophagy inhibitor 3-MA (5 mM) for 6 hours (*n* = 3 in each group). **(C, D)** Representative immunoblotting analysis (C) and quantification (D) of GSK3β in WT and *Atg5* KO HEK293T cells transfected with vector or *Optn*-OE (*n* = 3 in each group). **(E)** The quantification of LC3 immunoblotting analysis in *Optn* OE and *Optn* KD C2C12 cells at 4 days postdifferentiation (*n* = 3 in each group). **(F)** Representative immunofluorescence analysis of GFP-LC3, HA-OPTN, and FLAG-GSK3β in C2C12 cells transfected with GFP-LC3, HA-OPTN plasmids, and FLAG-GSK3β plasmids. Scale bars: 5 μm. **(G)** Co-immunoprecipitation analysis of LC3 and GSK3β in scramble shRNA or sh*Optn* TA muscle at 5 days postinjury. The immunoprecipitation analysis was performed in scramble shRNA or sh*Optn* TA muscle at 5 days postinjury incubated with anti-GSK3β antibody or nonspecific Rabbit IgG (control) to pulldown endogenous LC3. **(H)** Schematic illustration of the domain organization and molecular validation of mouse *Optn*-F188A and *Optn*-E481G point-mutant plasmids. **(I)** The quantification of OPTN, GSK3β, MYHC, and MYOG immunoblotting analysis in empty vector, WT-*Optn*, *Optn*-F188A, and *Optn*-E481G overexpressing C2C12 cells at 4 days postdifferentiation (*n* = 3 in each group). Data are presented as mean ± SEM. *P < 0.05 versus control. The underlying data for this figure can be found in **S1 Data**. The original blot for this figure can be found in **S1 Raw Image**. AAV, adeno-associated viral vector; CHX, cycloheximide; GSK3β, glycogen synthase kinase 3β; KD, knockdown; KO, knockout; LIR, LC3-interacting region; MYHC, myosin heavy chain; MYOG, myogenin; OE, overexpressing; OPTN, optineurin; SEM, standard error of the mean; shRNA, short hairpin RNA; TA, tibialis anterior; UBAN, ubiquitin-binding domain; 3-MA, 3-methyladenine.
(TIF)

S1 Table Primary antibodies used in this study. (DOCX)

S2 Table qRT-PCR primers used in this study. (DOCX)

S1 Data Contains underlying data for Figs 1A, 1F, 1G, 1I, 1J, 2D, 2E, 2G, 2H, 2J, 2K, 2M, 2N, 3A, 3B, 3C, 3D, 3F, 4A, 4B, 4C, 4D, 4E, 5I, 6A, 6B, 6D, 6F, 7B, 7D, and 7F and S1A, S1B, S3A, S3C, S3D, S4B, S4D, S4F, S4G, S5A, S5B, S6B, S6D, S8A, S8B, S8D, S8E, and S8I Figs. (XLSX)

S1 Raw Image Original blot contains Figs 1B, 1H, 2A, 2F, 2L, 3E, 3F, 4A, 4B, 4C, 4D, 4E, 5A, 5B, 5C, 5D, 5E, 5G, 6C, 6E, 7C, and 7E and S3A, S3D, S6A, S6C, S8C, and S8G Figs. (PDF)

## Abbreviations:

AAV: adeno-associated viral vector
ALS: amyotrophic lateral sclerosis
ANOVA: analysis of variance
APC: adenomatous polyposis coli protein
AXIN: axis inhibition protein
cDNA: complementary DNA
CHX: cycloheximide
CTX: cardiotoxin
CSA: cross-sectional fiber area
DEG: differentially expressed gene
DMD: Duchenne muscular dystrophy
DMEM: Dulbecco’s Modified Eagle medium
DVL2: disheveled-2
EdU: 5-ethynyl-20-deoxyuridine
eMYHC: embryonic myosin heavy chain
Fermt2: fermitin family homolog 2
FRAT: frequently rearranged in advanced T-cell lymphoma
GEO: Gene Expression Omnibus
GSK3β: glycogen synthase kinase 3β
HE: hematoxylin–eosin; H3, histone H3
KD: knockdown
LIR: LC3-interacting region
mdx: murine X-linked muscular dystrophy
MYHC: myosin heavy chain
Myod: myoblast determination protein
MYOG: myogenin
Myf5: myogenic factor 5
OE: overexpressing
OPTN: optineurin
Pax7: paired box 7
PVDF: polyvinylidene fluoride
SC: satellite cell
SDS-PAGE: sodium dodecyl sulfate-polyacrylamide gel electrophoresis
shOptn: short hairpin targeting OPTN
shRNA: short hairpin RNA
TA: tibialis anterior
TCF/LEF: T-cell factor/lymphoid enhancer factor
TCF4: transcription factor 4
UBAN: ubiquitin-binding domain
WGA: wheat germ agglutinin
WT: wild-type
3-MA: 3-methyladenine
