# Supplementary figures and images for: Optineurin promotes myogenesis during muscle regeneration in mice by autophagic degradation of GSK3β
Source: PLoS Biol. 2022 Apr 27;20(4):e3001619. doi: 10.1371/journal.pbio.3001619 (PMC9084533; doi:10.1371/journal.pbio.3001619)

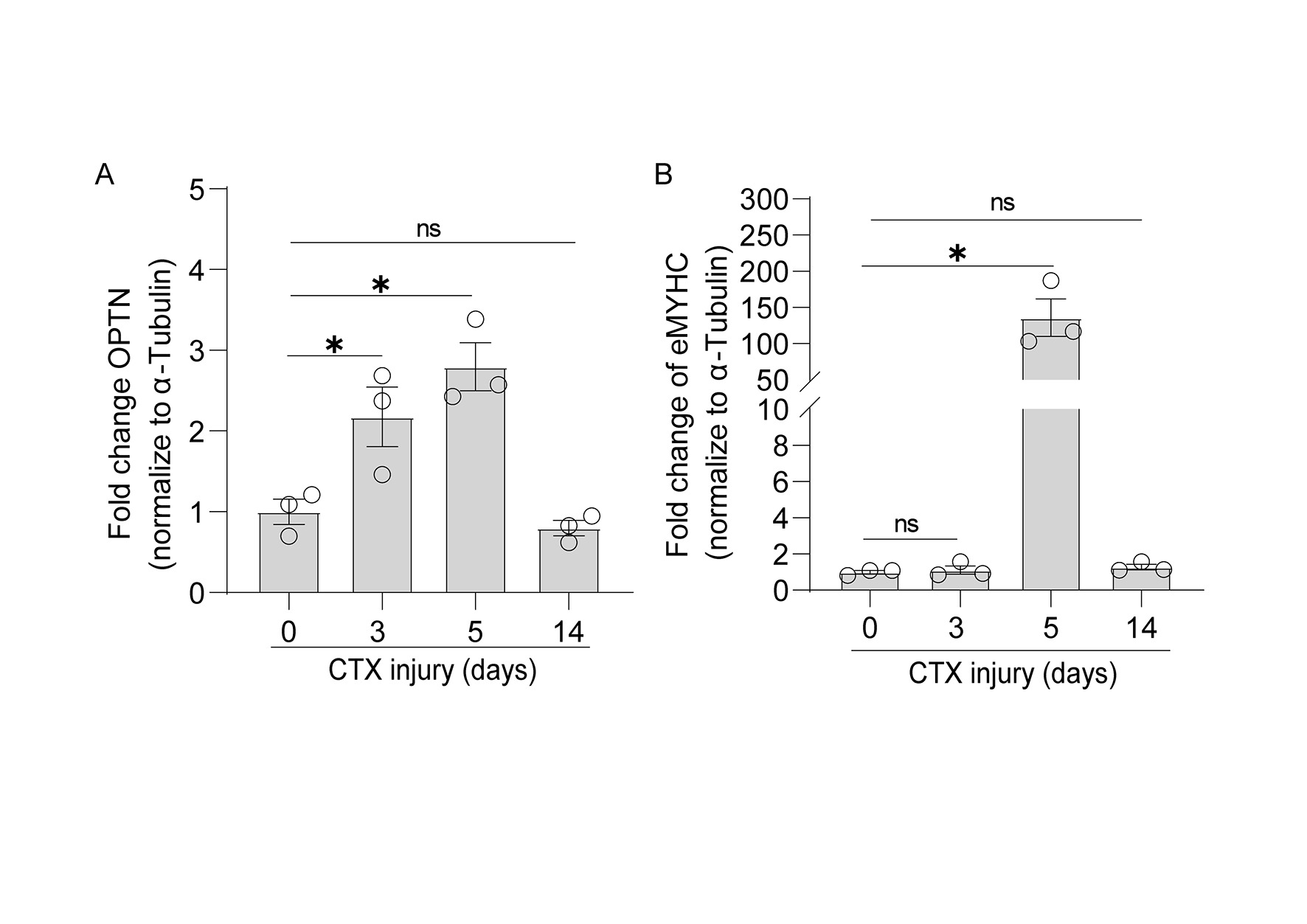

Supplement: S1 Fig — (A) The quantification of OPTN immunoblotting analysis in TA of WT mice at 0, 3, 5, and 14 days postinjury (n = 3 mice in each group). (B) The quantification of eMYHC immunoblotting analysis in TA of WT mice at 0, 3, 5, and 14 days postinjury (n = 3 mice in each group). Data are presented as mean ± SEM. *P < 0.05 versus control. The underlying data for this figure can be found in S1 Data. CTX, cardiotoxin; eMYHC, embryonic myosin heavy chain; OPTN, optineurin; SEM, standard error of the mean; TA, tibialis anterior; WT, wild-type. (TIF) [file pbio.3001619.s001.tif]

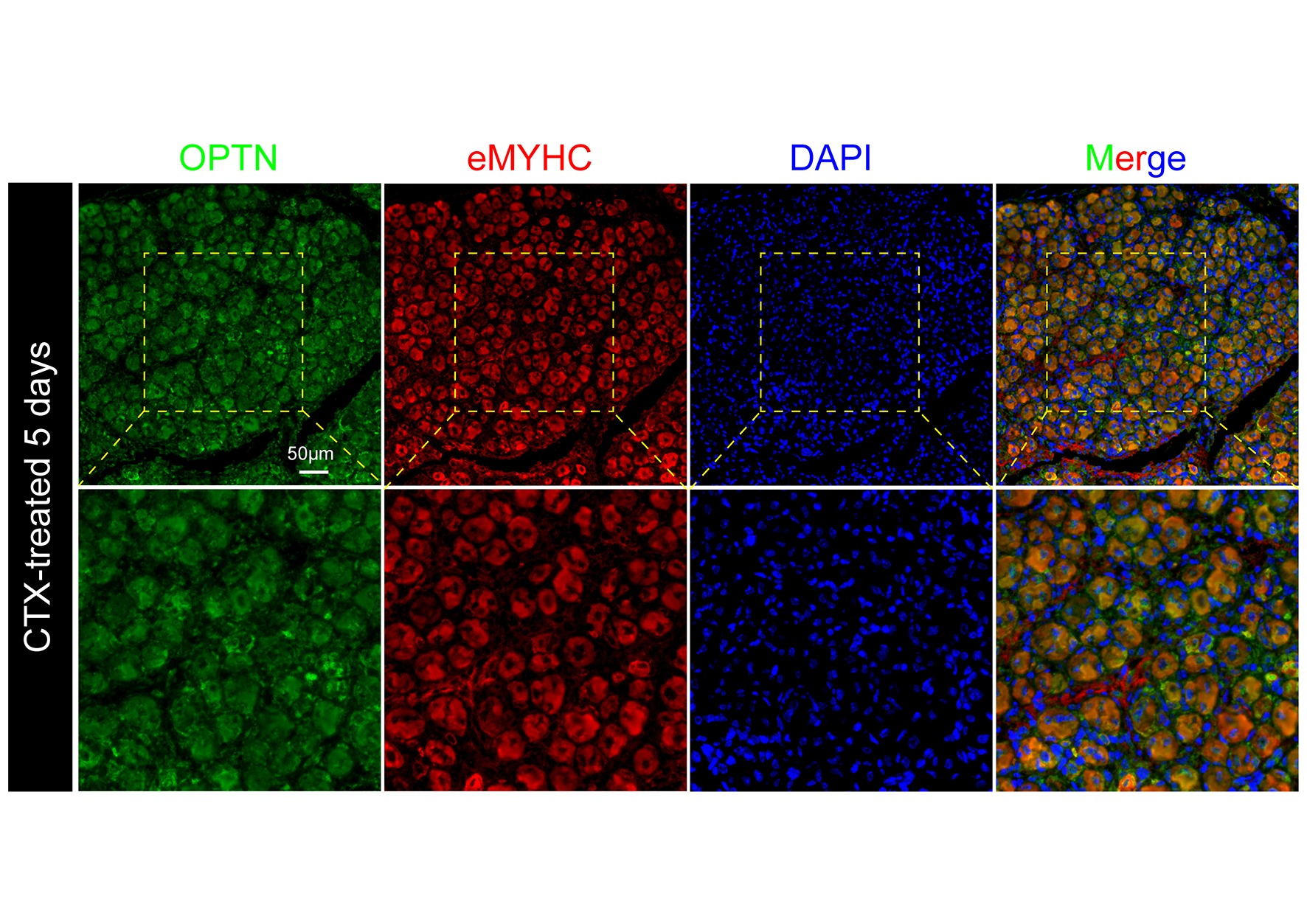

Supplement: S2 Fig — The OPTN, newly regenerated myofibers, and nucleus were stained with anti-OPTN antibody (red), anti-eMYHC antibody (green), and DAPI (blue), respectively. Scale bars: 50 um. eMYHC, embryonic myosin heavy chain; OPTN, optineurin; TA, tibialis anterior. (TIF) [file pbio.3001619.s002.tif]

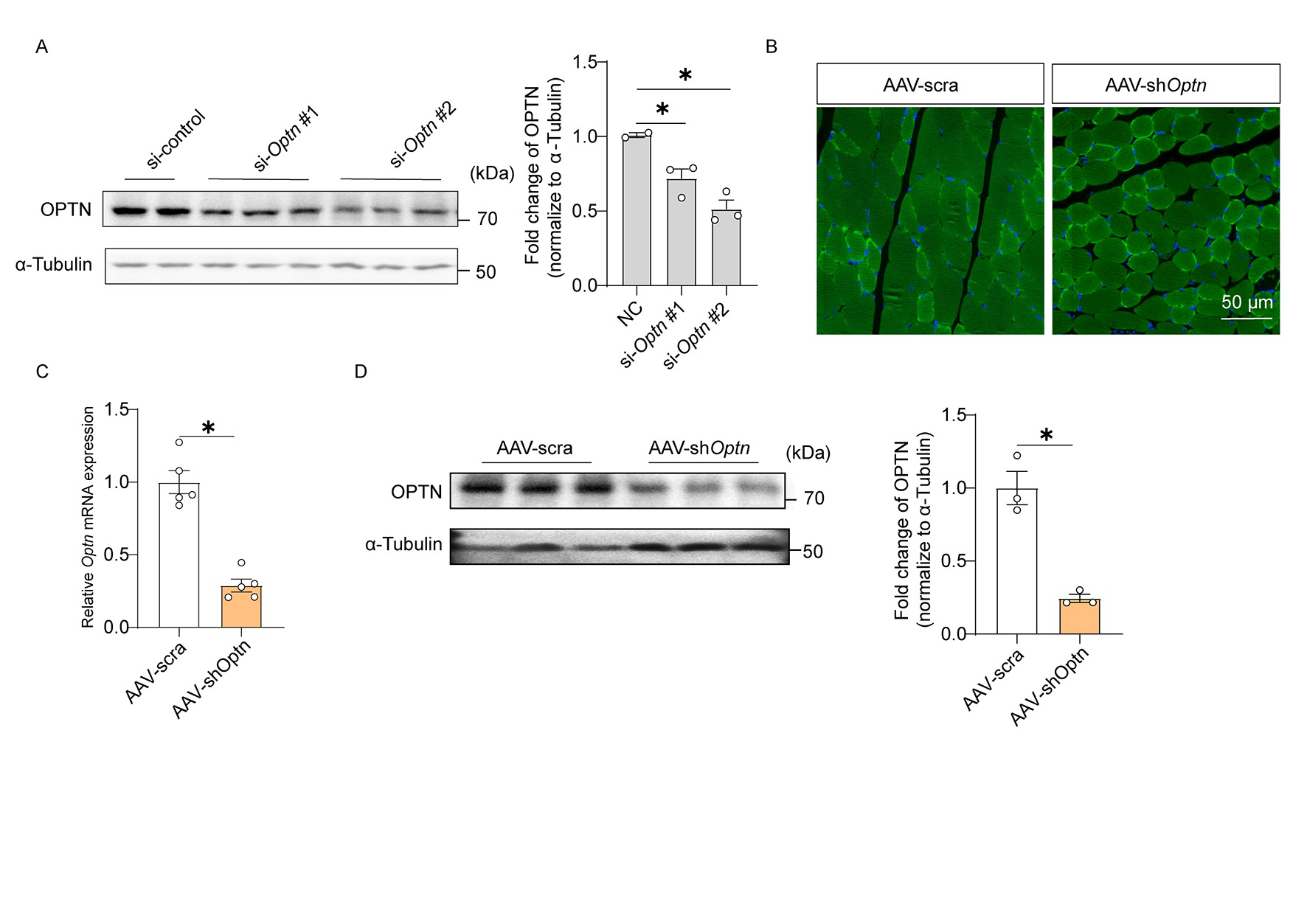

Supplement: S3 Fig — (A) Representative immunoblotting analysis (left panel) and quantification (right panel) of the OPTN in C2C12 myoblasts with si-control or si-Optn #1–2 transfection (n = 3 in each group). (B) Representative fluorescence image at 4 weeks postinjection of AAV containing scramble RNA or shOptn. (C) Quantification of Optn mRNA expression in TA muscle at 4 weeks postinjection of AAV containing scramble RNA or shOptn (n = 5 mice in each group). Representative immunoblotting analysis (left panel) and quantification (right panel) of the OPTN in TA muscle at 4 weeks postinjection of AAV containing scramble RNA or shOptn (n = 3 mice in each group). Data are presented as mean ± SEM. *P < 0.05 versus control. The underlying data for this figure can be found in S1 Data. The original blot for this figure can be found in S1 Raw Image. AAV, adeno-associated viral vector; KD, knockdown; OPTN, optineurin; SEM, standard error of the mean; shRNA, short hairpin RNA; TA, tibialis anterior. (TIF) [file pbio.3001619.s003.tif]

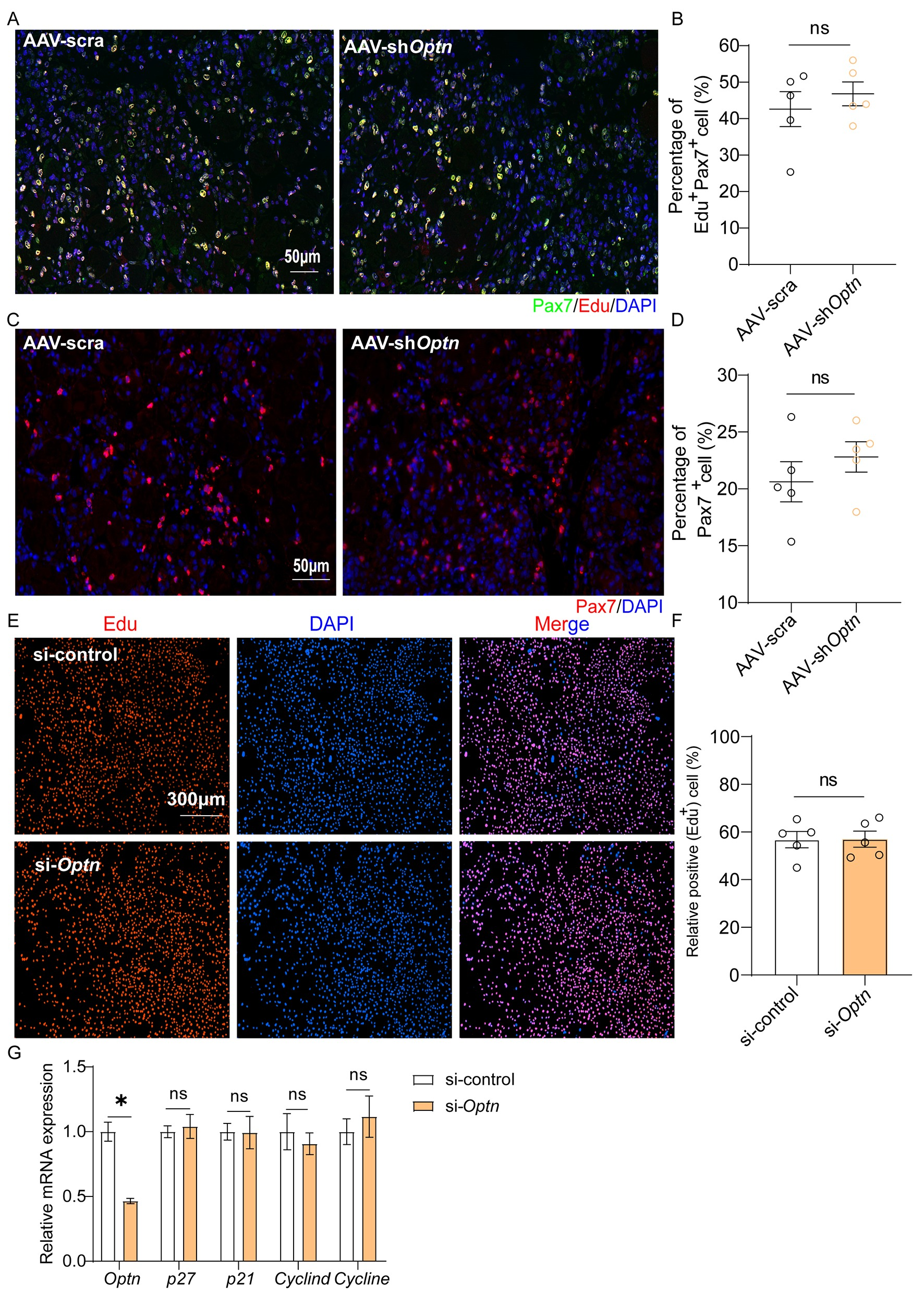

Supplement: S4 Fig — (A) Representative immunofluorescence staining of Pax7 (green), EdU (red), and DAPI (blue) in scramble shRNA or shOptn TA muscle at 3 days postinjury. Scale bar: 50 μm. (B) Quantification of the percentage of Pax7+EdU+ cells in scramble shRNA or shOptn TA muscle at 3 days postinjury (n = 5 mice in each group). (C) Representative immunofluorescence staining of Pax7 (red) and DAPI (blue) in scramble shRNA or shOptn TA muscle at 3 days postinjury. Scale bar: 50 μm. (D) Quantification of the percentage of Pax7+ cells in scramble shRNA or shOptn TA muscle at 3 days postinjury (n = 5 mice in each group). (E) Representative EdU and DAPI staining analysis in control (si-control) and Optn KD (si-Optn) C2C12 cells. si-control or si-Optn were transfected into C2C12 cells for 24 hours before staining analysis. Scale bar: 300 μm. (F) Quantification of the percentage of EdU-positive cells/total cells in control (si-control) and Optn KD (si-Optn) C2C12 cells (n = 5 in each group). si-control or si-Optn were transfected into C2C12 cells for 24 hours before staining analysis. (G) Representative mRNA expression analysis of cell proliferation–associated genes in C2C12 cells with si-control or si-Optn transfection (n = 6 in each group). Cells were collected after 24h transfection. Data are presented as mean ± SEM. *P < 0.05 versus control. The underlying data for this figure can be found in S1 Data. AAV, adeno-associated viral vector; EdU, 5-Ethynyl-2′-deoxyuridine; KD, knockdown; OPTN, optineurin; Pax7, paired box 7; SEM, standard error of the mean; shRNA, short hairpin RNA; TA, tibialis anterior. (TIF) [file pbio.3001619.s004.tif]

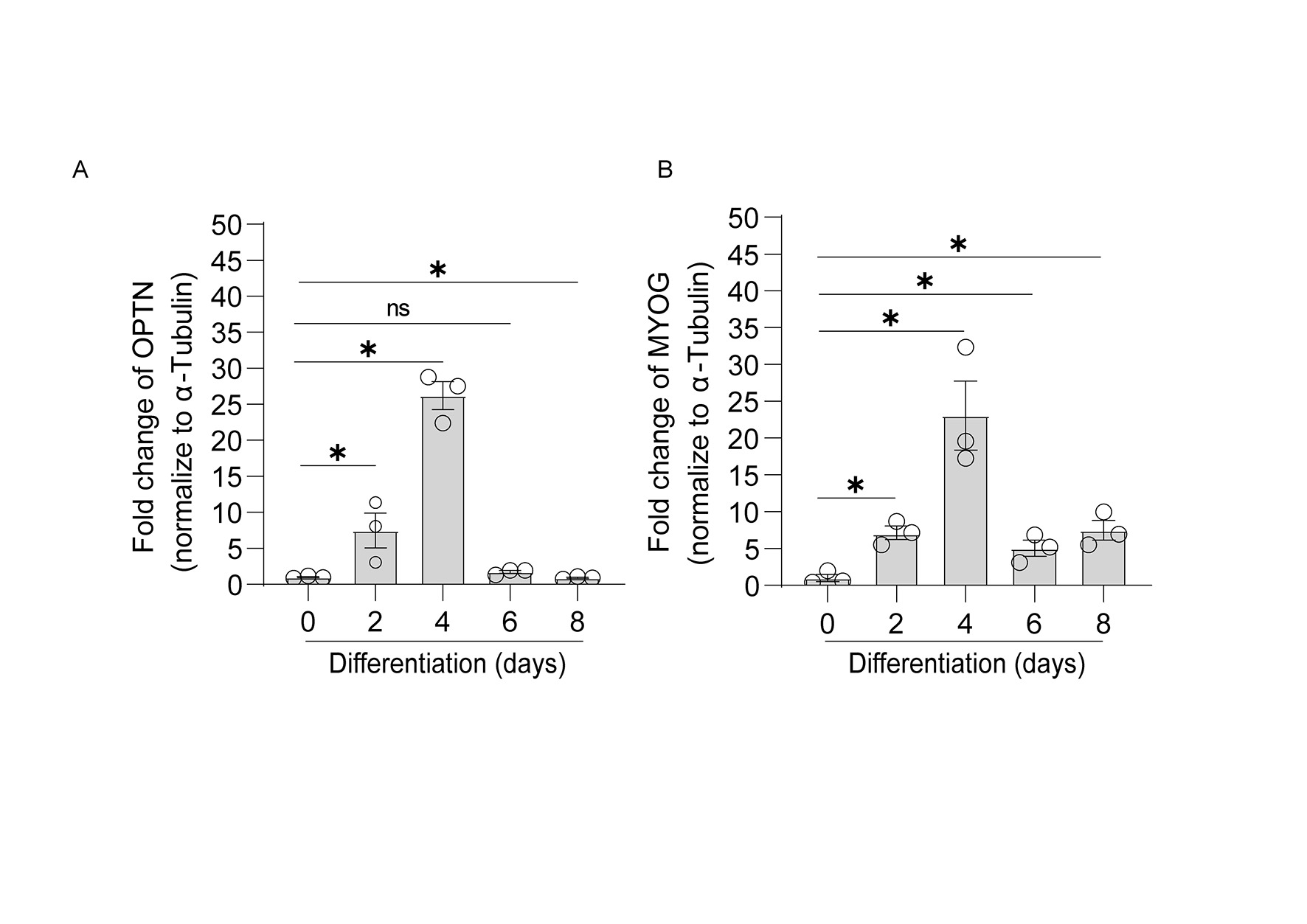

Supplement: S5 Fig — (A) The quantification of OPTN immunoblotting analysis in C2C12 cells during differentiation at the indicated time points (0, 2, 4, 6, and 8 days) (n = 3 in each group). (B) The quantification of MYOG immunoblotting analysis in C2C12 cells during differentiation at the indicated time points (0, 2, 4, 6, and 8 days) (n = 3 in each group). Data are presented as mean ± SEM. *P < 0.05 versus control. The underlying data for this figure can be found in S1 Data. MYOG, myogenin; Optn, optineurin; SEM, standard error of the mean. (TIF) [file pbio.3001619.s005.tif]

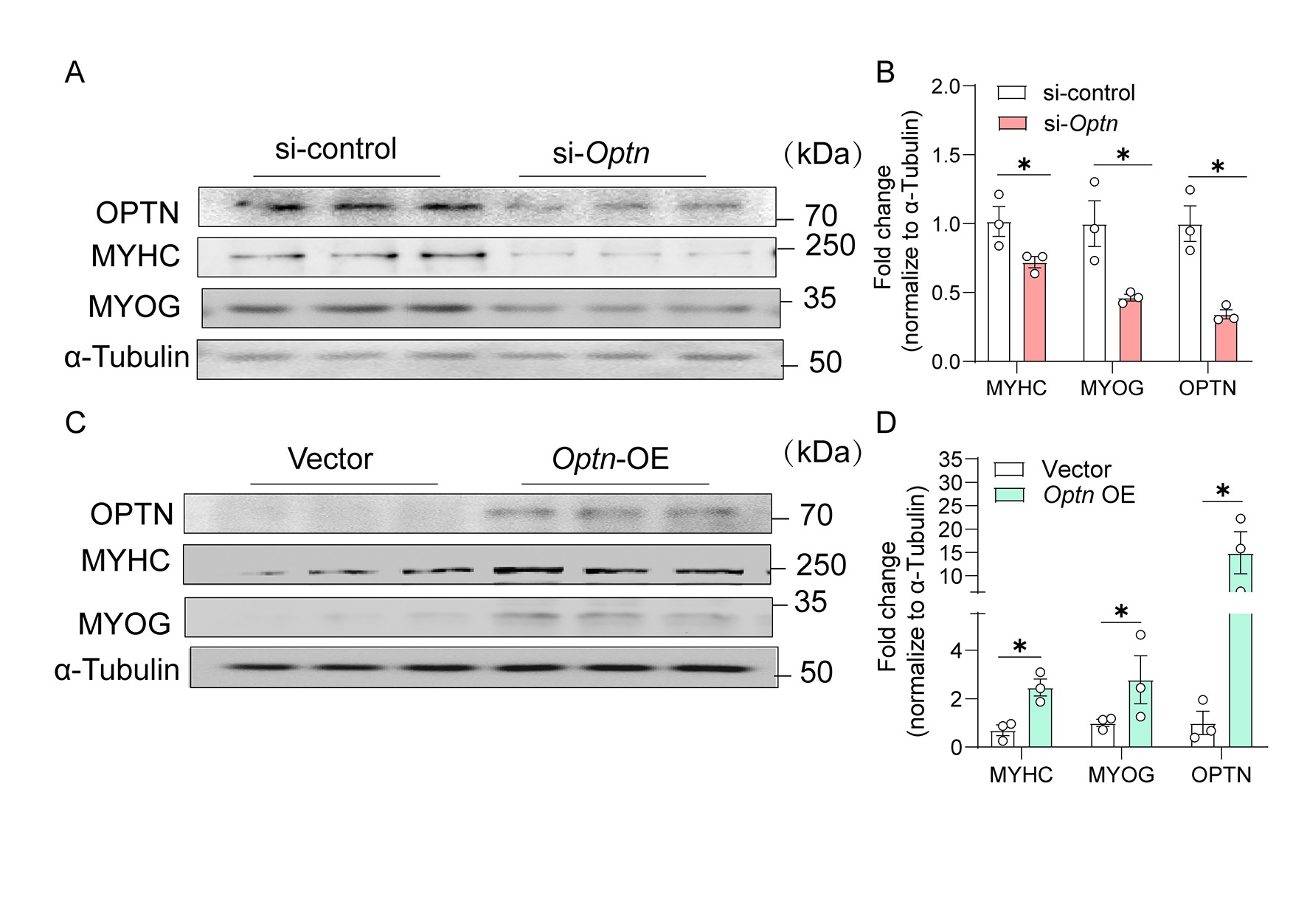

Supplement: S6 Fig — (A, B) Representative immunoblotting analysis (A) and quantification (B) of OPTN, MYHC, and MYOG in si-control or si-Optn C2C12 cells at 4 days postdifferentiation (n = 3 in each group). si-control or si-Optn were transfected into C2C12 cells for 48 hours before the initiation of differentiation. (C, D) Representative immunoblotting analysis (C) and quantification (D) of OPTN, MYHC and MYOG in control (empty vector) and Optn OE C2C12 cells at 4 days postdifferentiation (n = 3 in each group). The empty pcDNA 3.1-HA vector or pcDNA 3.1-HA-Optn vector were transfected into C2C12 cells for 48 hours before the initiation of differentiation. Cells were collected at 4 days postdifferentiation. Data are presented as mean ± SEM. *P < 0.05 versus control. The underlying data for this figure can be found in S1 Data. The original blot for this figure can be found in S1 Raw Image. MYOG, myogenin; MYHC, myosin heavy chain; OE, overexpressing; Optn, optineurin; SEM, standard error of the mean. (TIF) [file pbio.3001619.s006.tif]

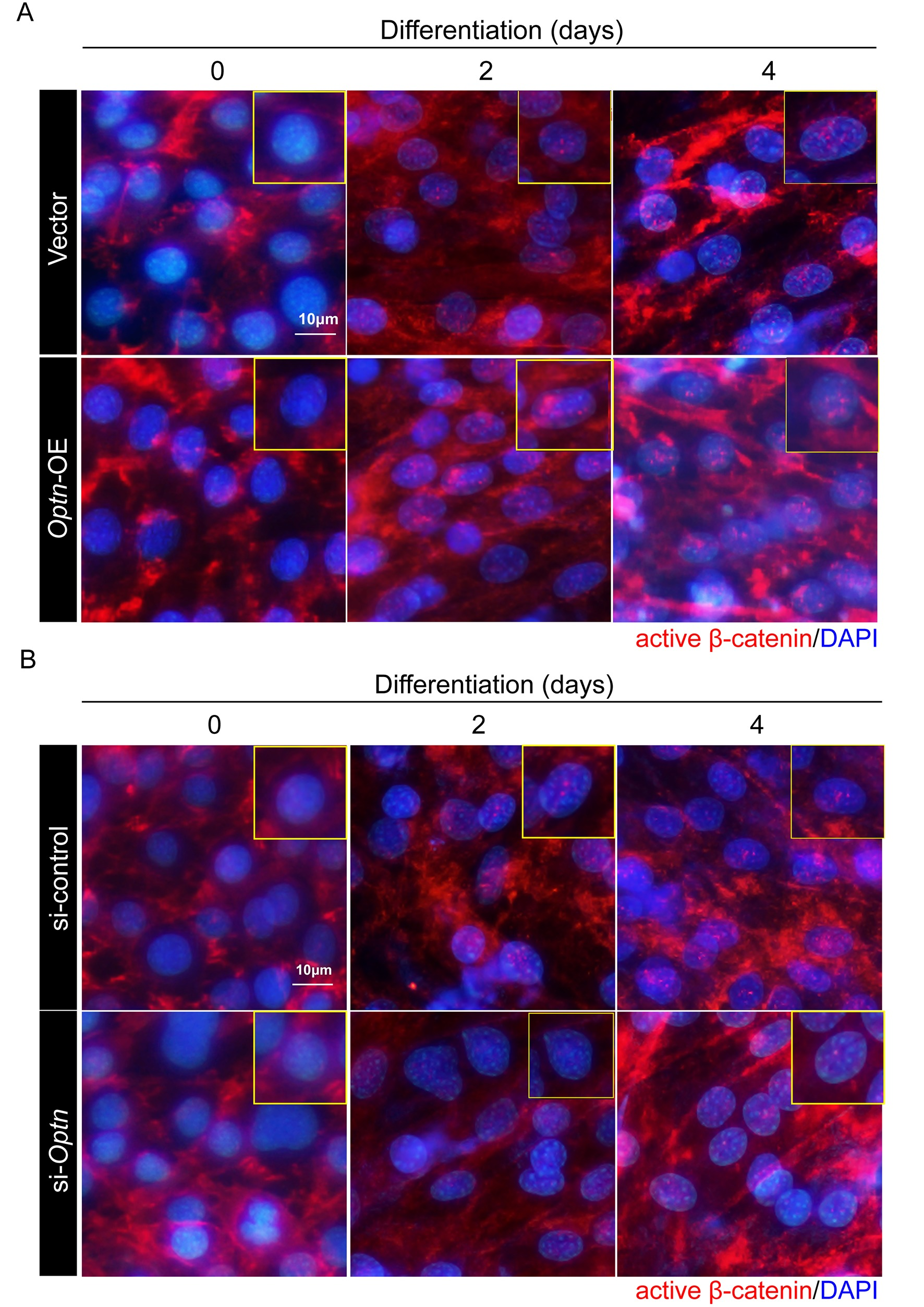

Supplement: S7 Fig — (A, B) Representative immunofluorescence analysis of active β-catenin in Optn OE (A) and Optn KD (B) C2C12 cells at 4 days postdifferentiation. Scale bar: 10 μm. KD, knockdown; OE, overexpressing; Optn, optineurin. (TIF) [file pbio.3001619.s007.tif]

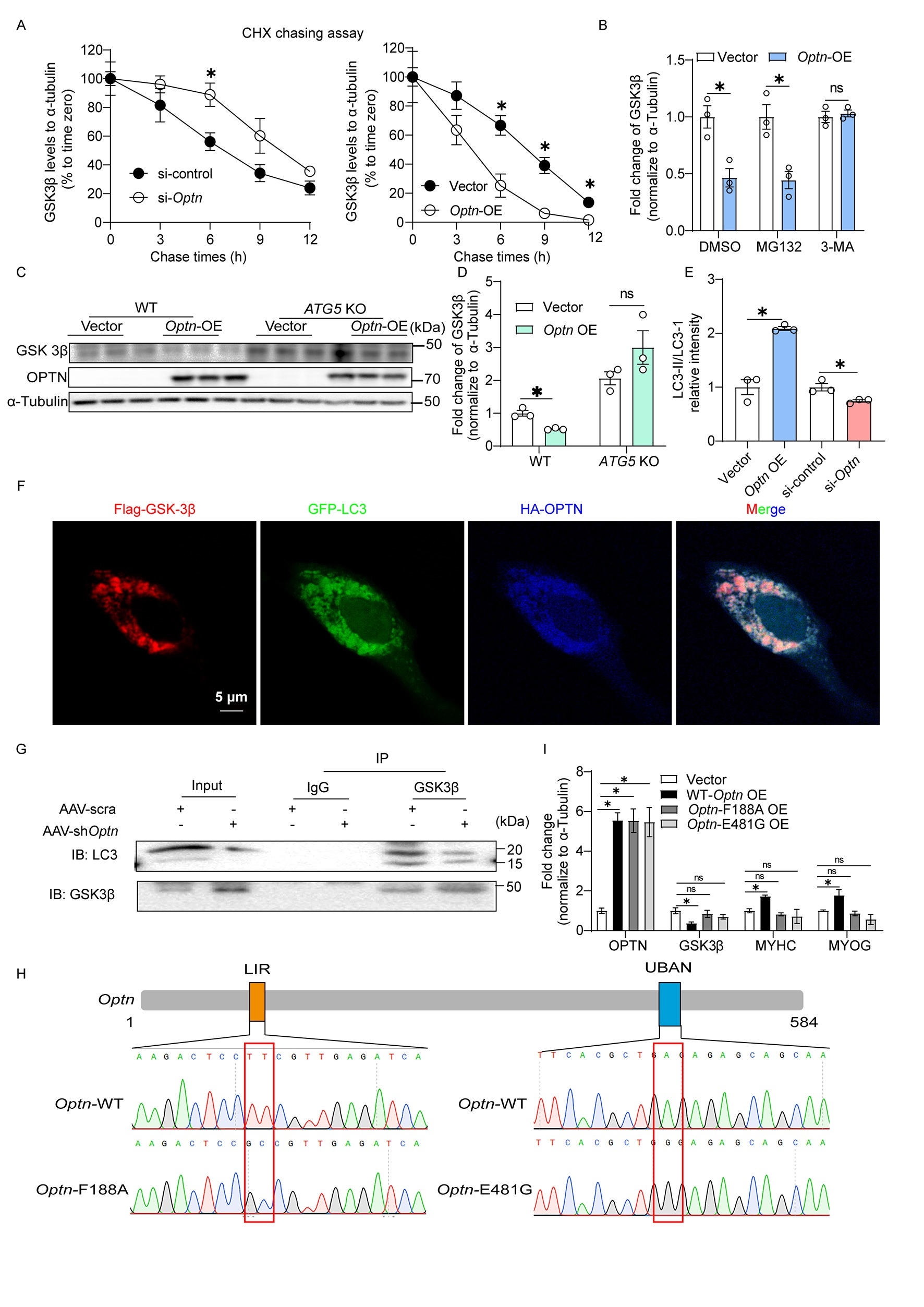

Supplement: S8 Fig — (A) The quantification of GSK3β immunoblotting analysis in Optn KD (left panel) and Optn OE (right panel) C2C12 cells at 4 days postdifferentiation and then treated with 50 μg/ml CHX at indicated time points (n = 3 in each group). (B) The quantification of GSK3β immunoblotting analysis in control (empty vector) or Optn-OE C2C12 cells at 4 days postdifferentiation and then treated with DMSO, the proteasome inhibitor MG132 (25 μM), or the autophagy inhibitor 3-MA (5 mM) for 6 hours (n = 3 in each group). (C, D) Representative immunoblotting analysis (C) and quantification (D) of GSK3β in WT and Atg5 KO HEK293T cells transfected with vector or Optn-OE (n = 3 in each group). (E) The quantification of LC3 immunoblotting analysis in Optn OE and Optn KD C2C12 cells at 4 days postdifferentiation (n = 3 in each group). (F) Representative immunofluorescence analysis of GFP-LC3, HA-OPTN, and FLAG-GSK3β in C2C12 cells transfected with GFP-LC3, HA-OPTN plasmids, and FLAG-GSK3β plasmids. Scale bars: 5 μm. (G) Co-immunoprecipitation analysis of LC3 and GSK3β in scramble shRNA or shOptn TA muscle at 5 days postinjury. The immunoprecipitation analysis was performed in scramble shRNA or shOptn TA muscle at 5 days postinjury incubated with anti-GSK3β antibody or nonspecific Rabbit IgG (control) to pulldown endogenous LC3. (H) Schematic illustration of the domain organization and molecular validation of mouse Optn-F188A and Optn-E481G point-mutant plasmids. (I) The quantification of OPTN, GSK3β, MYHC, and MYOG immunoblotting analysis in empty vector, WT-Optn, Optn-F188A, and Optn-E481G overexpressing C2C12 cells at 4 days postdifferentiation (n = 3 in each group). Data are presented as mean ± SEM. *P < 0.05 versus control. The underlying data for this figure can be found in S1 Data. The original blot for this figure can be found in S1 Raw Image. AAV, adeno-associated viral vector; CHX, cycloheximide; GSK3β, glycogen synthase kinase 3β; KD, knockdown; KO, knockout; LIR, LC3-interacting [file pbio.3001619.s008.tif]
