# Supplementary material for: Optineurin promotes myogenesis during muscle regeneration in mice by autophagic degradation of GSK3β
Source: PLoS Biol. 2022 Apr 27;20(4):e3001619. doi: 10.1371/journal.pbio.3001619 (PMC9084533; doi:10.1371/journal.pbio.3001619)
Supplement: S1 Table — (DOCX) [file pbio.3001619.s009.docx]

**S1 Table. Primary antibodies used in this study.**

| Antibody | Dilution (WB/IF/IP) | Source | Cat. No |
| --- | --- | --- | --- |
| OPTN | 1:1000/1:400 /1:50 | Cell Signaling Technology | 70928S |
| eMYHC | 1:1000/1:100/no | Bioss | bs-10905R |
| MYOG | 2 ug/mL/ 5 ug/mL/no | Novus Biologicals | NB100-56510 |
| α-TUBULIN | 1:4000/no/no | Proteintech | 11224-1-AP |
| PAX7 | no/1:100/no | Proteintech | 20570-1-AP |
| MYHC | 1:2000/1:100/no | R&D systems | MAB4470 |
| Active β-catenin | 1:1000/1:100/no | Cell Signaling Technology | 8814S |
| H3 | 1:4000/no/no | Abcam | ab1791 |
| DVL2 | 1:1000/no/no | Proteintech | 12037-1-AP |
| GSK3β | 1:1000/no/4 ug | Proteintech | 22104-1-AP |
| AXIN | 1:1000/no/no | Santa Cruz | sc-293190 |
| APC | 1:1000/no/no | Santa Cruz | sc-9998 |
| LC3 | 1:1000/no/3 ug | Proteintech | 14600-1-AP |
| Goat anti-rabbit (HRP) | 1:10000/no/no | Abbkine | A21020 |
| Goat anti-mouse (HRP) | 1:10000/no/no | Abbkine | A21021 |
| Goat anti-rabbit (Alexa Fluor 546) | no/1:1000/no | Invitrogen | A-11035 |
| Goat anti-mouse (Alexa Fluor 546) | no/1:1000/no | Invitrogen | A-11003 |
| Goat anti-rabbit (Alexa Fluor 350) | no/1:100/no | Beyotime Biotechnology | A0412 |
| Goat anti-mouse (FITC) | no/1:1000/no | Transgene | HS211-01 |

**Table S2**: qRT-PCR primers used in this study.

| Gene | Forward | Reverse | Size (bp) | Accession Number |
| --- | --- | --- | --- | --- |
| *Myc* | TAGTGCTGCATGAGGAGACA | CTCCACAGACACCACATCAA | 92 | NM_001177353.1 |
| *Ccnd3* | GCGTGCAAAAGGAGATCAAGCC | CCAGGTAGTTCATAGCCAGAGG | 117 | NM_001081636.1 |
| *Twist2* | CAGCAAGATCCAGACGCTCAAG | ACACGGAGAAGGCGTAGCTGAG | 140 | NM_007855.3 |
| *Mycn* | TGTGTCTGTTCCAGCTACTGCC | CATCTTCCTCCTCGTCATCCTC | 146 | NM_008709.3 |
| *Optn* | TGTCAGGCTCTGGAGAGGAA | GTCTTGGCCTGCTCCATCTT | 137 | NM_001356487.1 |
| *p27* | TCAAACGTGAGAGTGTCTAACG | CCGGGCCGAAGAGATTTCTG | 103 | NM_009875.4 |
| *p21* | CCTGGTGATGTCCGACCTG | CCATGAGCGCATCGCAATC | 103 | NM_001111099.2 |
| *CyclinE* | GTGGCTCCGACCTTTCAGTC | CACAGTCTTGTCAATCTTGGCA | 101 | NM_007633.2 |
| *CyclinD* | GCGTACCCTGACACCAATCTC | CTCCTCTTCGCACTTCTGCTC | 183 | NM_001379248.1 |
