# Supplementary material for: Optineurin promotes myogenesis during muscle regeneration in mice by autophagic degradation of GSK3β
Source: PLoS Biol. 2022 Apr 27;20(4):e3001619. doi: 10.1371/journal.pbio.3001619 (PMC9084533; doi:10.1371/journal.pbio.3001619)
Supplement: S2 Table — (DOCX) [file pbio.3001619.s010.docx]

**S2 Table**. **qRT-PCR primers used in this study.**

| Gene | Forward | Reverse | Size (bp) | Accession Number |
| --- | --- | --- | --- | --- |
| *Myc* | TAGTGCTGCATGAGGAGACA | CTCCACAGACACCACATCAA | 92 | NM_001177353.1 |
| *Ccnd3* | GCGTGCAAAAGGAGATCAAGCC | CCAGGTAGTTCATAGCCAGAGG | 117 | NM_001081636.1 |
| *Twist2* | CAGCAAGATCCAGACGCTCAAG | ACACGGAGAAGGCGTAGCTGAG | 140 | NM_007855.3 |
| *Mycn* | TGTGTCTGTTCCAGCTACTGCC | CATCTTCCTCCTCGTCATCCTC | 146 | NM_008709.3 |
| *Optn* | TGTCAGGCTCTGGAGAGGAA | GTCTTGGCCTGCTCCATCTT | 137 | NM_001356487.1 |
| *p27* | TCAAACGTGAGAGTGTCTAACG | CCGGGCCGAAGAGATTTCTG | 103 | NM_009875.4 |
| *p21* | CCTGGTGATGTCCGACCTG | CCATGAGCGCATCGCAATC | 103 | NM_001111099.2 |
| *CyclinE* | GTGGCTCCGACCTTTCAGTC | CACAGTCTTGTCAATCTTGGCA | 101 | NM_007633.2 |
| *CyclinD* | GCGTACCCTGACACCAATCTC | CTCCTCTTCGCACTTCTGCTC | 183 | NM_001379248.1 |
