# Supplementary material for: Optineurin promotes myogenesis during muscle regeneration in mice by autophagic degradation of GSK3β
Source: PLoS Biol. 2022 Apr 27;20(4):e3001619. doi: 10.1371/journal.pbio.3001619 (PMC9084533; doi:10.1371/journal.pbio.3001619)

Figure 1

Fig. 1B

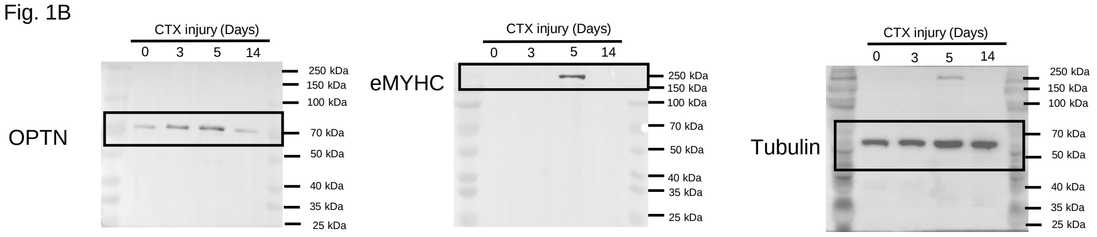

Fig. 1H

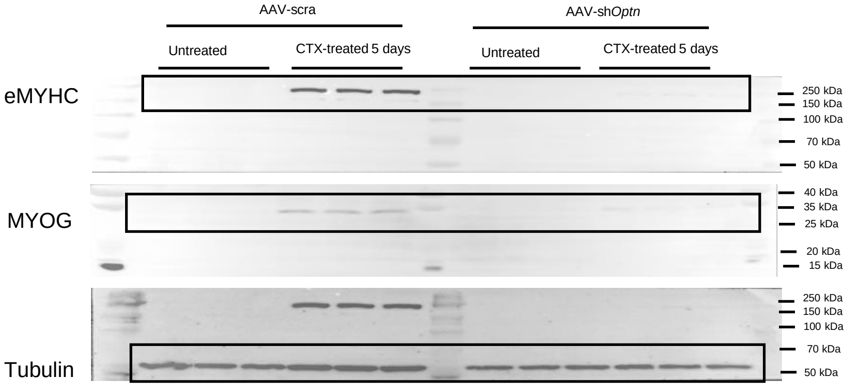

Figure 2

Fig. 2A

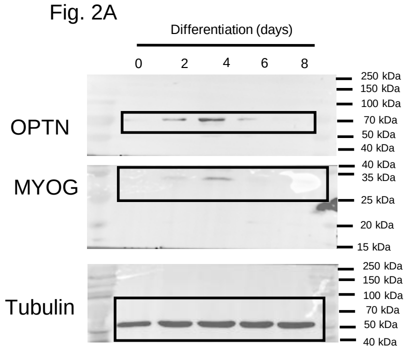

Fig. 2F

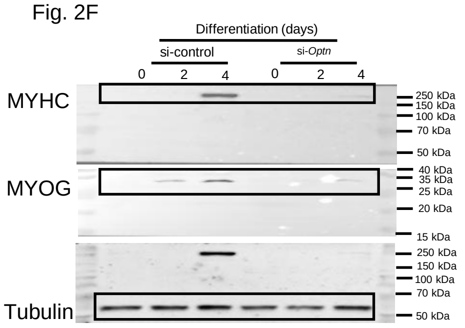

Fig. 2L

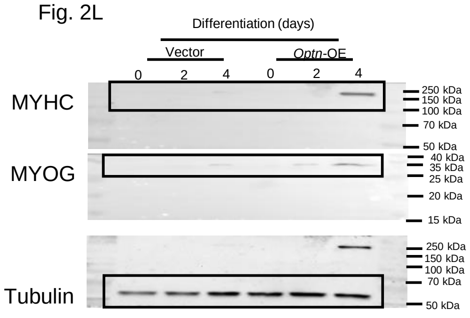

Figure 3

Fig. 3E-F

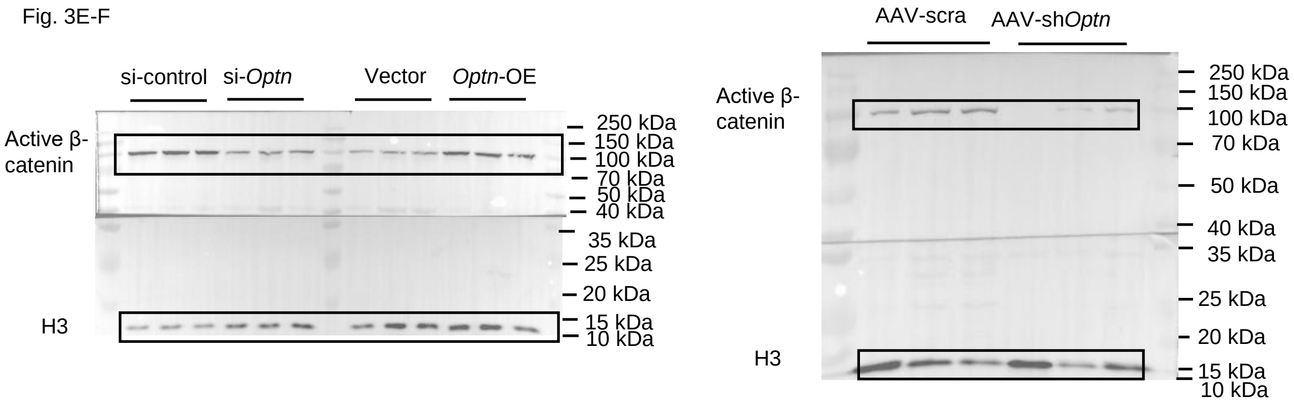

Figure 4

Fig. 4A-B

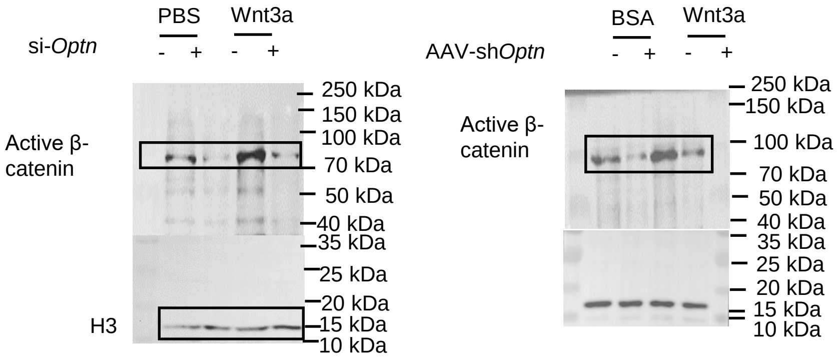

Figure 4

Fig. 4C-D

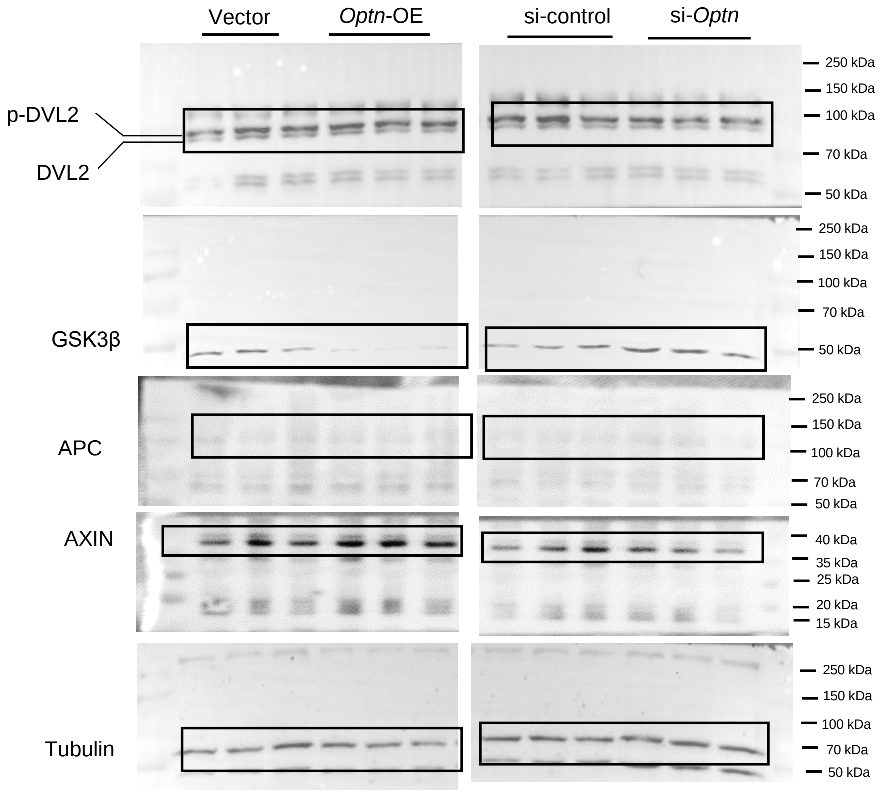

Fig. 4E

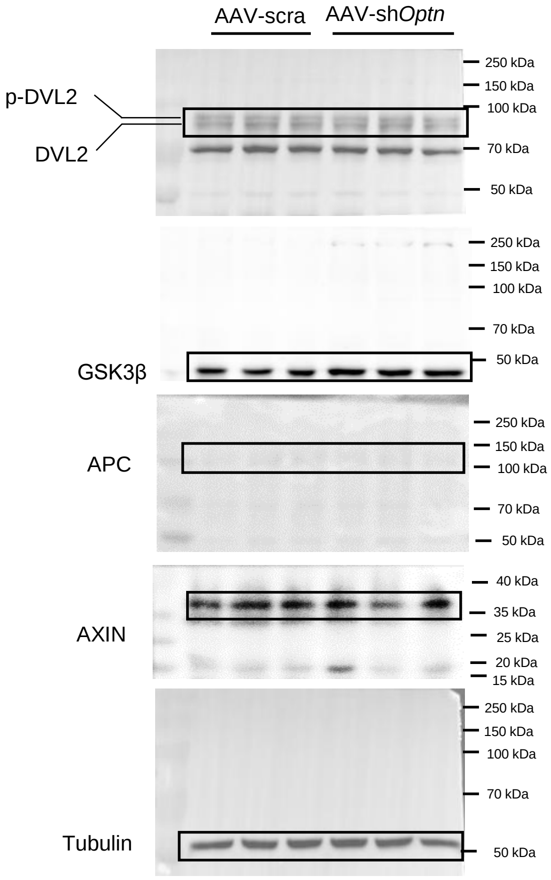

Figure 5

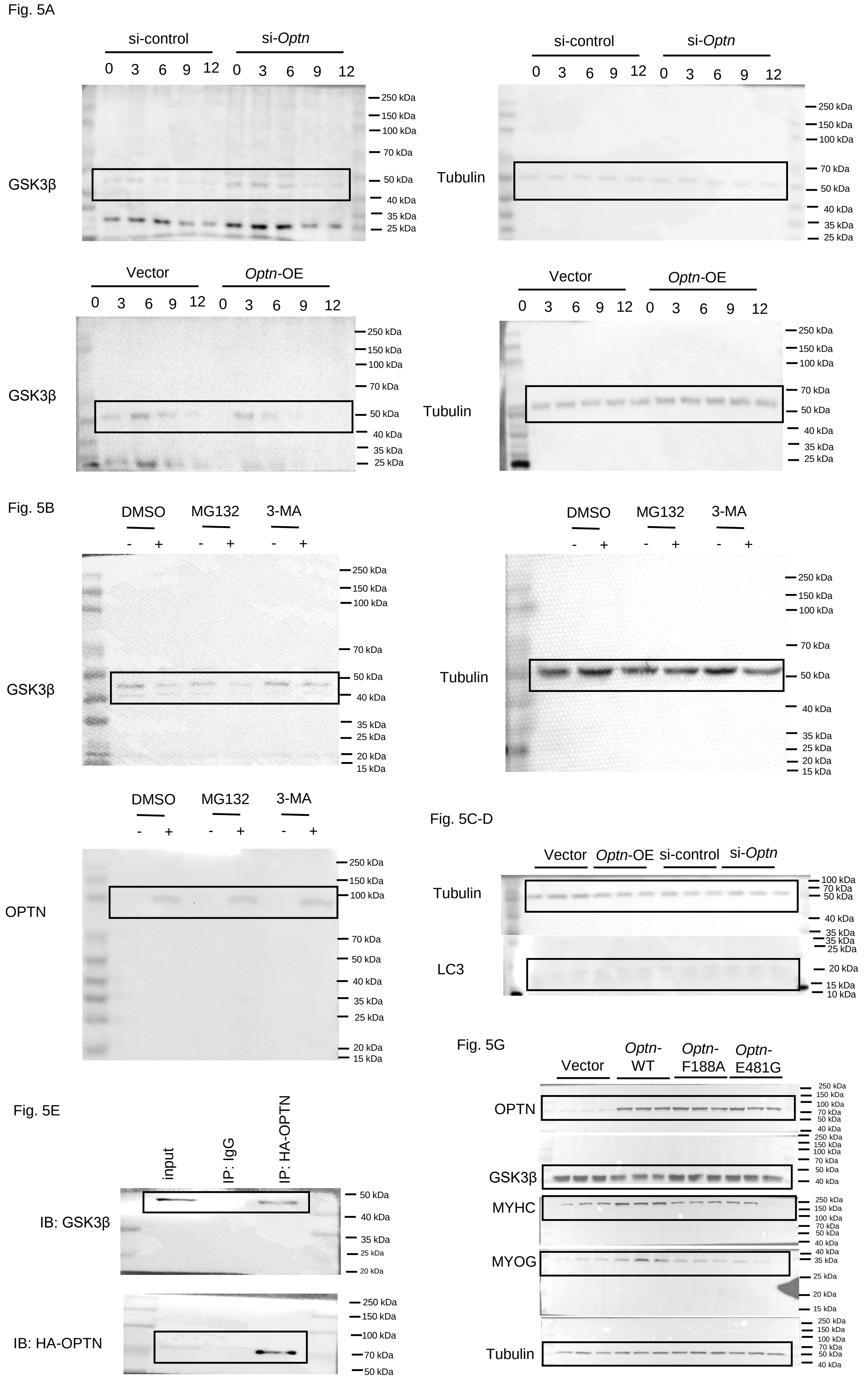

Fig. 5A
si-control
si-Optn
0 3 6 9 12 0 3 6 9 12
GSK3β
Tubulin
250 kDa
150 kDa
100 kDa
70 kDa
50 kDa
40 kDa
35 kDa
25 kDa
Vector
Optn-OE
Fig. 5B
DMSO
MG132
3-MA
- + - + - +
20 kDa
15 kDa
OPTN
Fig. 5C-D
Vector
Optn-OE
si-control
si-Optn
LC3
10 kDa
Fig. 5E
input
IP: IgG
IP: HA-OPTN
IB: GSK3β
IB: HA-OPTN
Fig. 5G
Vector
Optn-WT
Optn-F188A
Optn-E481G
MYHC
MYOG

Figure 6

Fig. 6C

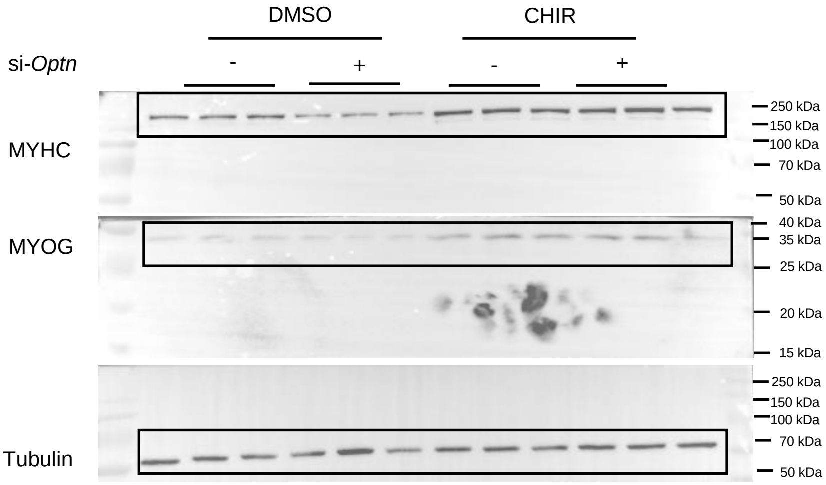

Fig. 6E

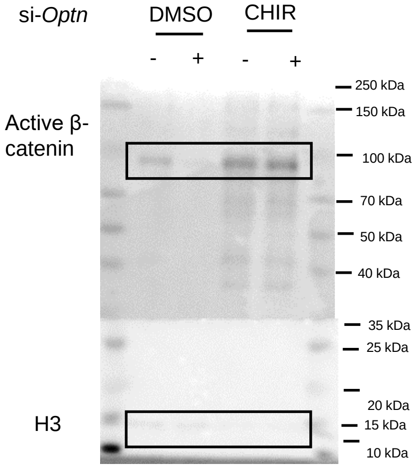

Figure 7

Fig. 7C

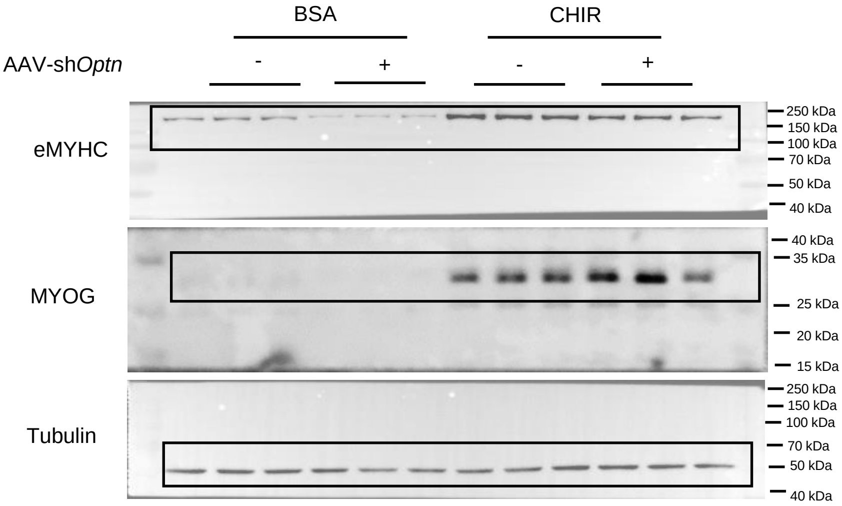

Fig. 7E

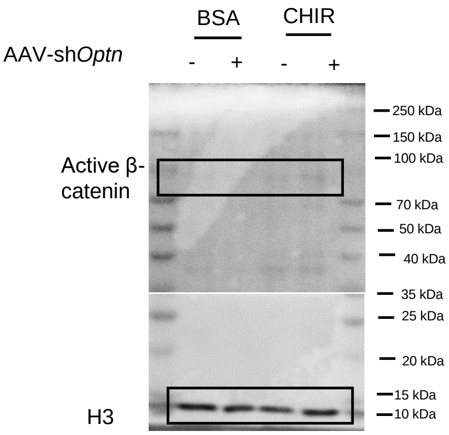

Figure S3

Fig. S3A

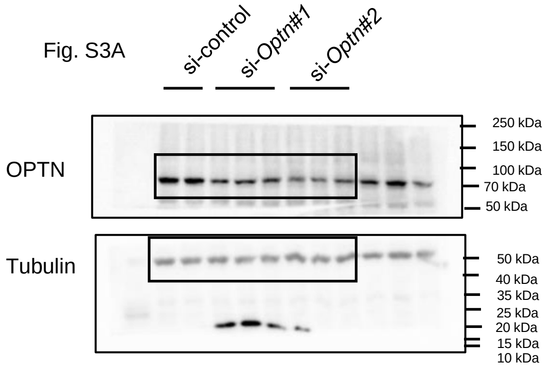

Fig. S3D

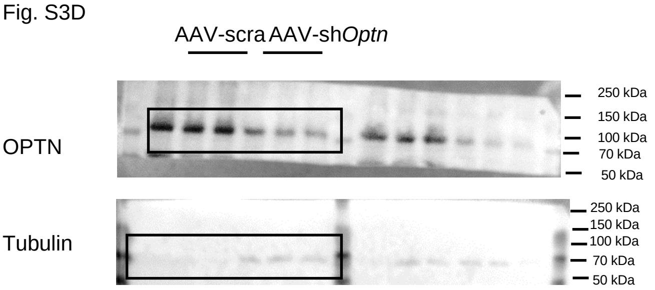

Figure S6

Fig. S6A

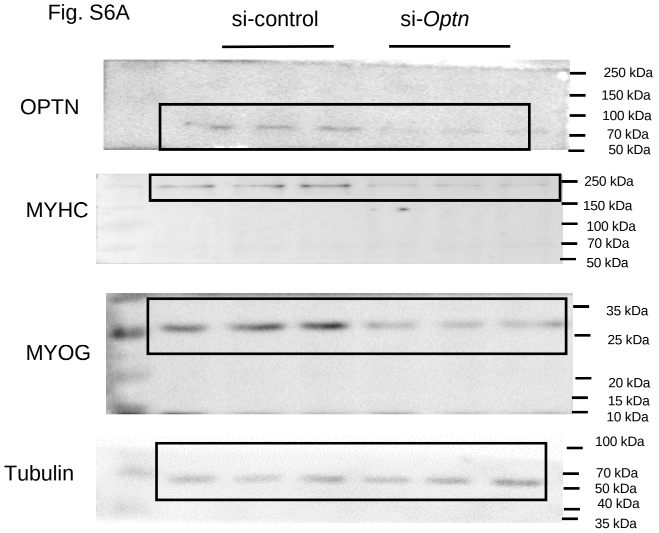

Fig. S6C

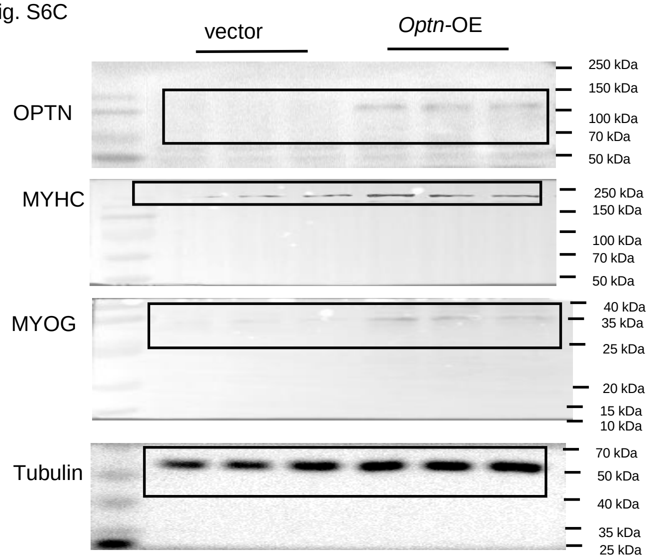

Figure S8

Fig. S8C

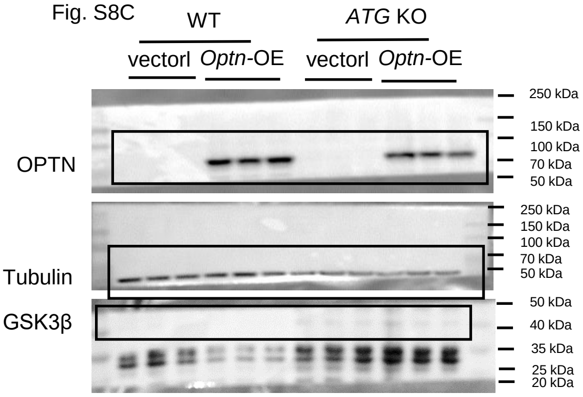

Fig. S8G

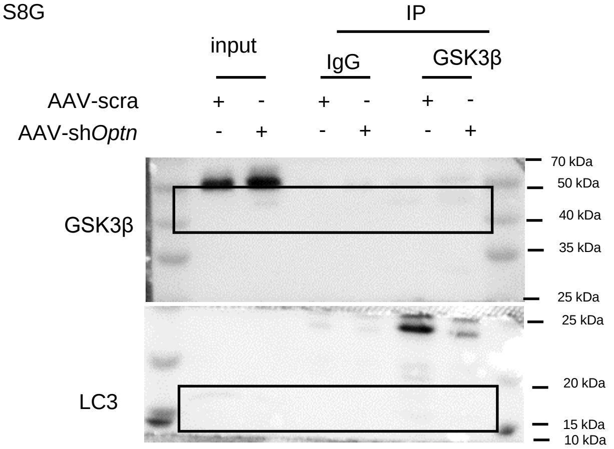

Supplement: S1 Raw Image — (PDF) [file pbio.3001619.s012.pdf]
